# NSUN2-mediated m5C modification of HIV-1 RNA enables evasion of RIG‑I‑dependent innate immunity

**DOI:** 10.1371/journal.ppat.1014611

**Published:** 2026-09-25

**Authors:** Yuebo Xu, Mingyang Li, Yingying Song, Weili Kong, Jingjing Song, Jiali Zhang, Xinmu Xu, Haoyu Ma, Shun Yu, Shuliang Chen, Cong Zeng

**Affiliations:** 1 Key Laboratory of Medical Molecular Virology (MOE/NHC/CAMS), Shanghai Institute of Infectious Disease and Biosecurity, School of Basic Medical Sciences, Shanghai Medical College, Fudan University, Shanghai, China; 2 Institute of Medical Virology, Taikang Medical School (School of Basic Medical Sciences), Wuhan University, Wuhan, Hubei, China; Sun Yat-Sen University, CHINA

## Abstract

Recently, 5-methylcytosine (m5C) modification has been identified in HIV-1 genomic RNA. However, the functional role of this RNA modification in the antiviral innate immune response remains unclear. Here, we demonstrate that m5C modification of HIV-1 genomic RNA enables the virus to evade the type I interferon (IFN-I)-mediated antiviral response, thereby promoting viral replication. Depletion of NSUN2 in viral-producing cells significantly reduced m5C modification of HIV-1 RNA, leading to progeny viruses that are more susceptible to innate immune detection and consequently displaying attenuated replication. Furthermore, *in vitro*-transcribed m5C-modified RNA exhibited a reduced ability to induce IFN-I production relative to unmodified RNA. Additionally, m5C-modified RNA displayed markedly impaired binding to RIG-I compared with unmodified RNA. Collectively, our findings reveal that HIV-1 utilizes m5C modification of viral genomic RNA as a strategy to escape RIG-I-mediated immune recognition, thereby facilitating efficient viral replication.

## Author summary

HIV-1 remains a major global health challenge, in part because the virus has evolved clever ways to avoid being detected by the human immune system. In our study, we set out to understand how HIV-1 evades the body’s first line of defense, known as innate immunity. We discovered that HIV-1 hijacks a human protein called NSUN2 to add a chemical mark called m5C to its own genomic RNA. This mark hides the viral RNA from RIG-I, which normally recognizes foreign RNA and triggers an antiviral response. By masking itself this way, HIV-1 can replicate more efficiently and establish infection. Our findings reveal a new strategy HIV-1 uses to evade the immune system, linking RNA chemical modifications to viral escape. This knowledge not only deepens our understanding of how HIV-1 survives in the body but also points to potential new targets for developing treatments that could help the immune system recognize and fight the virus.

## Introduction

Acquired immune deficiency syndrome (AIDS), caused by human immunodeficiency virus (HIV-1), remains a persistent global public health challenge despite the success of antiretroviral therapy (ART) in suppressing viral replication and delaying disease progression [1,2]. A critical unresolved issue in HIV-1 research is the insufficient innate immune clearance observed in ART-treated patients, which is closely linked to the ability of HIV-1 to evade host surveillance mediated by pattern recognition receptor (PRR) [3]. As a retrovirus, the life cycle of HIV-1 includes RNA intermediates that should be recognized by RIG-I and MDA5 [4–7]. These key PRRs initiate type I interferon (IFN-I) production and antiviral responses upon sensing viral RNA [8–11]. However, the IFN-I induction is extremely weak during HIV-1 infection, and the molecular mechanisms by which HIV-1 evades host innate immunity remain incompletely understood [12,13].

RNA chemical modifications have emerged as important regulators of viral-host interactions, and various modification shape immune responses during viral infections [14–18]. Among these modifications, N6-methyladenosine (m6A) is the most prevalent internal RNA modification and has been widely investigated [19,20]. m6A modification on HIV-1 RNA can suppress IFN-I induction in differentiated monocytic cells and primary macrophages by promoting *IFNB1* mRNA degradation and regulating nuclear export [20,21]. Similarly, 2’-O-ribose methylation of the viral RNA cap, catalyzed by host methyltransferase-like protein 1 (MTr1), enables influenza viruses and HIV-1 to evade PRR recognition. This modification shields viral RNA from detection by host PRRs, thereby promoting viral replication [22–24]. Another well-characterized modification is 5-methylcytosine (m5C), which plays critical roles in diverse aspects of RNA processing, including tRNA stability, rRNA assembly, and mRNA translation [25–28]. The m5C modification is dynamically regulated by methyltransferases known as writers, including NSUN2, effector proteins referred to as readers that mediate its functional outcomes, and demethylases known as erasers that remove the modification [26,29,30].

Notably, while the roles of m6A and 2’-O-methylation in HIV-1 immune evasion are increasingly defined, the function of m5C modification in HIV-1 infection remains poorly understood, especially potential involvement in evasion from PRRs-mediated RNA sensing. A prior epitranscriptomic study demonstrated via purified HIV-1 genomic RNA that viral transcripts bear more epitranscriptomic modifications than average cellular mRNA, with m5C and 2′O-methyl modifications being particularly prevalent, and identified NSUN2 as the primary methyltransferase responsible for m5C deposition on HIV-1 RNAs [31]. Meanwhile, accumulating evidence has established broad immunoregulatory functions for RNA m5C across viral infections. For instance, m5C modification of hepatitis B virus (HBV) RNA inhibits RIG-I binding and suppresses IFN production, highlighting an important role of this modification in viral immune escape [32]. Another study showed that depletion of NSUN2 leads to an enhanced type I interferon response, which significantly inhibits replication of a wide range of RNA and DNA viruses in vitro [33]. Despite these relevant observations, inconsistent results exist regarding viral m5C deposition: a later study using rigorous bisulfite sequencing detected no verifiable m5C sites within the RNA of SARS-CoV-2, HIV-1 or MLV [34]. To date, no direct evidence has linked HIV-1 RNA m5C modification to PRRs-mediated immune escape. These conflicting published data and outstanding knowledge gaps highlight the importance of elucidating the function of m5C in HIV-host interactions.

In this study, we report that HIV-1 hijacks the host m5C methyltransferase NSUN2 to mediate m5C modification of viral genomic RNA. This modification directly blocks RIG-I binding to viral RNA, thereby suppressing RIG-I-mediated IFN-I production and downstream antiviral signaling. We further demonstrate that NSUN2-mediated m5C modification enhances HIV-1 infectivity and replication by abrogating innate immune recognition. Depletion of NSUN2 or loss of m5C modification restores RIG-I signaling during HIV-1 infection. This study identifies a novel mechanism by which HIV-1 exploits host RNA methylation machinery to evade innate immune surveillance, expanding our understanding of HIV-host interactions and highlighting the NSUN2-m5C RNA-RIG-I axis as a potential therapeutic target for restoring innate antiviral immunity against HIV-1.

## Results

### NSUN2 enhances the infectivity of HIV-1 progeny viruses

It has been reported that NSUN2 serves as the primary writer responsible for m5C modification on HIV-1 genomic RNA [32]. However, the functional importance of NSUN2-mediated m5C modification during HIV-1 infection remains poorly defined.

**Fig 1:**
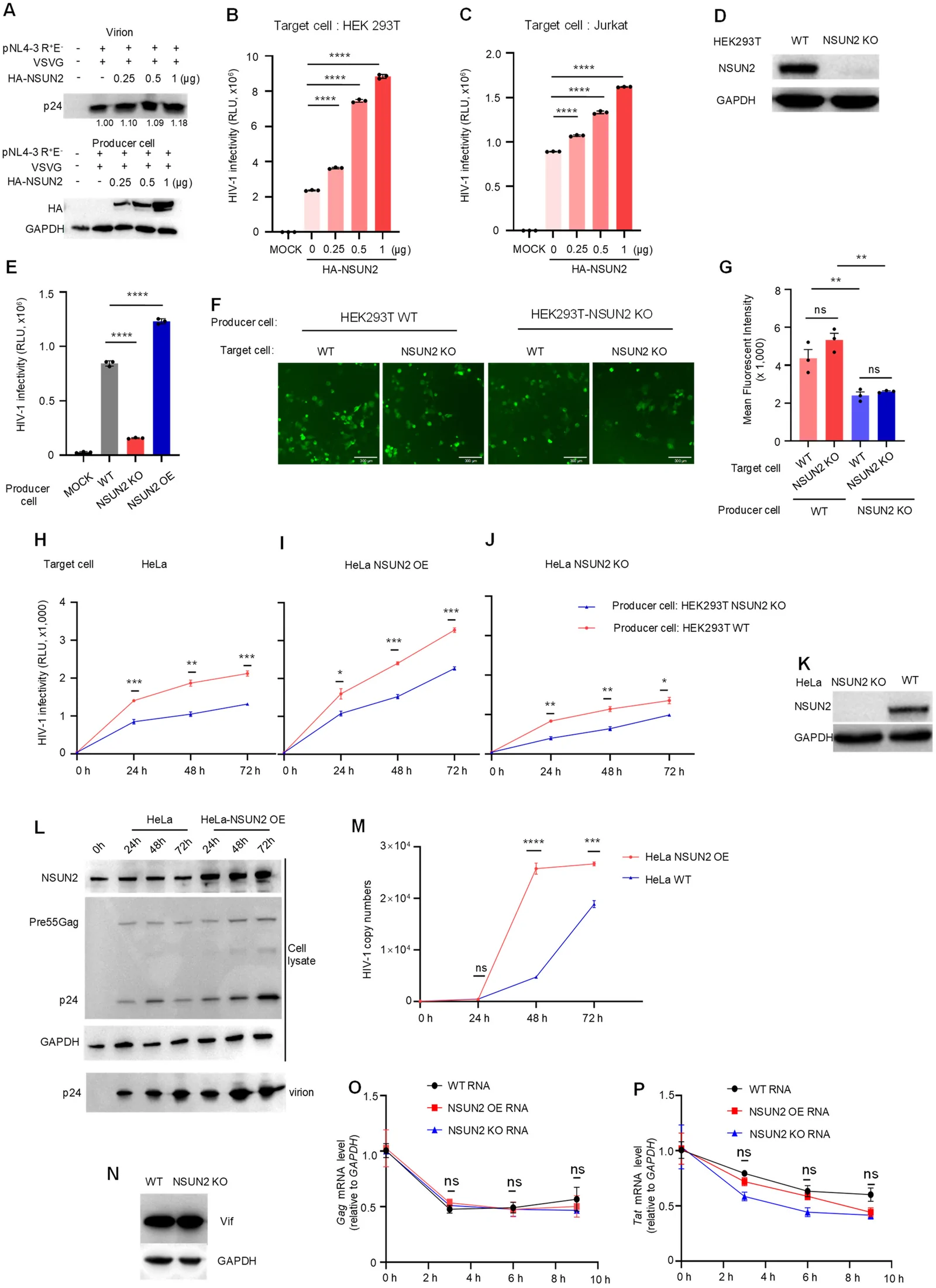
NSUN2 enhances the infectivity of the progeny viruses. (A) HEK293T cells were co-transfected with pNL4–3-R ⁺ E^−^-luc and VSVG plasmids for HIV-1 pseudovirus production, together with increasing doses of NSUN2 expression plasmid. Cells and virions were harvested for Western blot analysis. (B-C) Normalized HIV-1 pseudoviruses from (A) were used to infect HEK293T and Jurkat cells to measure viral infectivity. Cells were lysed at 36 h post-infection and subjected to firefly luciferase activity assay. (D) Western blot analysis of NSUN2 expression in WT and NSUN2 KO HEK293T cells. (E) Infectivity of HIV-1 pseudoviruses produced from WT, NSUN2-overexpressing (OE), or NSUN2 KO cells. (F-G) VSVG-pseudotyped HIV-1-GFP viruses were produced in WT or NSUN2 KO cells and used to infect WT or NSUN2 KO cells (n = 3). Representative images were shown with uniform background correction to reduce non-specific background signals (F), and infected cells were quantified by flow cytometry (G). (H-J) HIV-1 pseudoviruses produced from WT or NSUN2 KO cells were used to infect VSVG-transfected WT HeLa (H), NSUN2-overexpressing HeLa (I), and NSUN2 KO HeLa (J) cells. Cells were harvested at the indicated time points to measure firefly luciferase activity. (K) Western blot analysis of NSUN2 expression in WT and NSUN2 KO HeLa cells. (L) Western blot analysis of viral p24 protein in cell lysates and culture supernatants collected from VSV-G transfected WT HeLa and NSUN2-overexpressing HeLa cells. (M) Absolute qPCR quantification of HIV-1 Gag transcripts in infected WT HeLa and NSUN2-overexpressing HeLa cells treated as described in Fig 1L. (N) Western blot detection of Vif protein in HEK293T cells infected with pseudotyped HIV-1 derived from NSUN2 WT or KO producer cells. (O, P) Actinomycin D chase assays measuring HIV-1 RNA stability in WT, NSUN2 KO and NSUN2 OE HEK293T cells transfected with pNL4–3. Transcription was blocked by actinomycin D, Gag and Tat mRNA was quantified at serial time points via RT-PCR. Data are representative of three independent experiments and analyzed by two-tailed unpaired *t*-test or one-way ANOVA. Values are presented as means ± SEM (n = 3). NS, not significant (P > 0.05), *P < 0.05, **P < 0.01, ****P < 0.0001.

To investigate the role of NSUN2 in HIV-1 virion production and progeny virus infectivity, we overexpressed NSUN2 in a dose‑dependent manner in HEK293T cells during the production of firefly luciferase‑based HIV-1 pseudovirus. Progeny viruses were harvested and normalized by p24 levels prior to infectivity assays. We found that NSUN2 overexpression in virus‑producing cells did not affect virion production (Fig 1A), but significantly enhanced the infectivity of progeny viruses in both HEK293T and Jurkat cells (Fig 1B–C). To validate these observations, we generated NSUN2 knockout (KO) HEK293T cells (Fig 1D). When NSUN2 KO cells were used as virus producer cells, the infectivity of progeny viruses was remarkably reduced compared with viruses derived from wild‑type (WT) HEK293T cells or NSUN2‑overexpressing cells (NSUN2 OE) (Fig 1E). Together, these complementary overexpression and knockout results demonstrate that NSUN2 abundance in producer cells positively controls the infectivity of newly assembled HIV-1 particles without altering total virion output.

To further confirm the effects of NSUN2 in virus‑producing cells and target cells, we produced single‑cycle HIV-1G‑FP viruses in either WT or NSUN2 KO HEK293T cells, then used these viruses to infect both WT and NSUN2 KO target cells. Fluorescence microscopy revealed that progeny viruses derived from NSUN2 KO cells exhibited markedly lower infectivity than those from WT cells, regardless of whether the target cells were WT or NSUN2 KO (Fig 1F). GFP‑positive populations were subsequently quantified by flow cytometry (Fig 1G). To mimic multiple‑cycle HIV-1 replication, WT, NSUN2 KO (Fig 1K), or NSUN2 OE HeLa target cells were transfected with VSV‑G and infected with the corresponding pseudoviruses. Cells were collected for firefly luciferase detection to reflect HIV-1 replication. Data showed that viruses derived from WT cells (red lines) replicated much faster than those from NSUN2 KO cells (blue lines). Notably, HIV-1 replication efficiency correlated positively with NSUN2 expression levels in target cells, being lowest in NSUN2 KO cells and highest in NSUN2‑overexpressing cells (Fig 1H–J). Culture supernatants collected from HeLa WT and NSUN2 OE cells infected with viruses generated from WT HEK293T producer cells were subjected to quantification of viral RNA and p24 protein to assess viral output. Consistently, NSUN2 overexpression in HeLa cells significantly boosted pseudotyped HIV-1 production (Fig 1L and 1M). These results collectively demonstrate that NSUN2 positively regulates HIV-1 replication.

Given that m5C modification is known to modulate RNA stability, splicing and nuclear export, we tested whether the attenuated infectivity of HIV-1 produced in NSUN2 KO cells resulted from accelerated viral RNA degradation or suppressed translation. To assess whether NSUN2 loss alters HIV-1 RNA stability, we performed actinomycin D chase assays to monitor viral RNA decay kinetics. Briefly, HEK293T WT, NSUN2 KO and NSUN2-overexpressing cells were transfected with the HIV-1 pNL4–3 proviral plasmid. At 24 h post-transfection, actinomycin D was added to block nascent transcription, and total RNA was harvested at indicated time points for quantitative detection of viral mRNA. No significant differences in HIV-1 RNA degradation rates were observed across all three cell groups, indicating that NSUN2-dependent m5C modification exerts negligible effects on viral RNA stability (Fig 1O and 1P). Notably, we only quantified the RNA stability of HIV-1 gag and tat transcripts in this work. The decay kinetics of other RNA species such as total host RNAs and virion-packaged tRNAs were not characterized here, and we cannot fully exclude the possibility that altered stability of these untested RNAs may contribute to the observed reduction in viral infectivity. We next investigated whether translational efficiency of viral transcripts was compromised upon NSUN2 deletion, with particular focus on the m5C-modified vif transcript [32]. Western blot analysis showed that NSUN2 knockout in virus-producing cells did not alter the expression level of Vif protein in HIV-1 infected cells (Fig 1N). Collectively, these data rule out altered viral RNA stability and impaired translation as potential causes of diminished viral infectivity.

Together, these results demonstrate that NSUN2 expression level is critical for the infectivity of progeny HIV-1 and plays an essential role in promoting viral replication.

### NSUN2 in viral producer cells suppresses HIV-1 progeny virus-induced type I IFN

Because NSUN2 altered progeny virus infectivity without markedly changing virion production or viral RNA stability, we next asked whether this phenotype reflected altered recognition by the innate immune system. It has been reported that m6A and 2’-O-methylation of HIV-1 RNA enables the virus to evade antiviral innate immunity by suppressing IFN-I induction [35,36]. However, whether NSUN2-mediated m5C modification of viral genomic RNA provides a similar immune evasion strategy during HIV-1 infection remains unclear.

**Fig 2:**
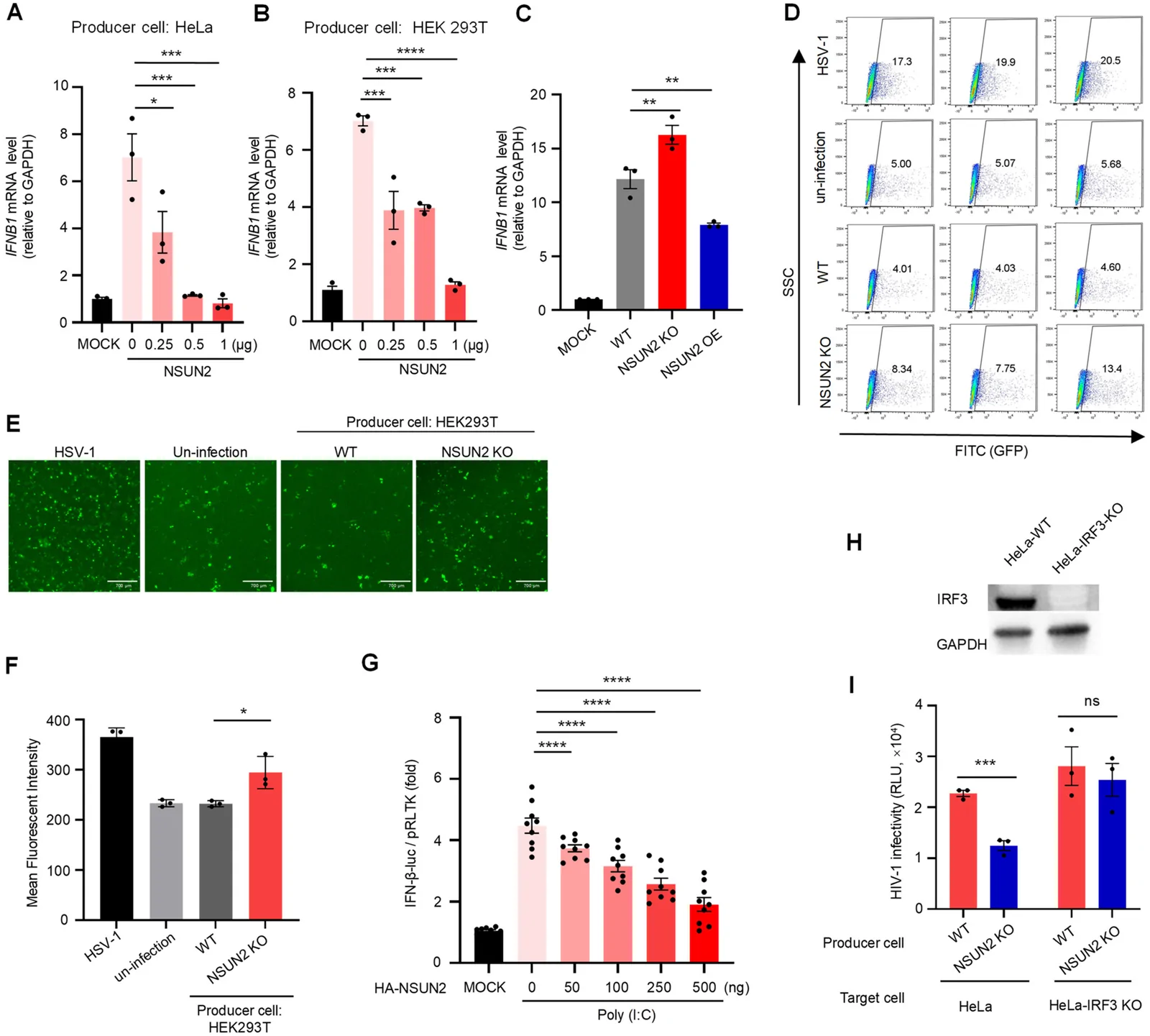
NSUN2 in viral producer cells suppresses HIV-1 progeny virus-induced type I IFN. (A-B) Measurement of *IFNB1* mRNA level in HEK293T cells infected with HIV-1 pseudoviruses produced from NSUN2-overexpressing HeLa cells (A) or HEK293T cells (B). (C) Measurement of *IFNB1* mRNA level in HEK293T cells infected with HIV-1 pseudoviruses produced from WT, NSUN2-overexpressing, and NSUN2 KO cells. (D-F) Flow cytometry analysis of the IFN-β-GFP reporter system activated by HIV-1 pseudoviruses as described in (C) (n = 3). (G) Dual-luciferase assay analyzing IFN-β promoter activity (IFN-β-Luc) in HEK293T cells transfected with empty vector or increasing amounts (0, 0.05, 0.1, 0.25, 0.5 μg) of NSUN2, followed by poly(I:C)-mediated activation of the IFN-β pathway. (H) Western blot validation of IRF3 protein ablation in IRF3-KO HeLa cell line. (I) Measurement of HIV-1 infectivity in WT HeLa cells and IRF3-KO HeLa cells. VSV-G pseudotyped HIV-1 were produced from WT and NSUN2 KO cells. Data are representative of three independent experiments and analyzed by two-tailed unpaired *t*-test or one-way ANOVA. Values are presented as means ± SEM (n = 3). NS, not significant (P > 0.05), *P < 0.05, **P < 0.01, ***P < 0.001, ****P < 0.0001.

To examine the role of NSUN2 in IFN‑I induction during HIV-1 infection, we overexpressed NSUN2 in a dose‑dependent manner in HeLa or HEK293T producer cells during the generation of VSV‑G‑pseudotyped HIV-1. Progeny viruses were harvested and used to infect HEK293T target cells. Total RNA was extracted from infected cells and analyzed by RT‑PCR. We found that NSUN2 expression in virus-producing cells markedly reduced *IFNB1* mRNA levels in target cells induced by progeny virus infection (Fig 2A–B). Conversely, progeny viruses derived from NSUN2 knockout (KO) producer cells elicited significantly higher *IFNB1* mRNA expression (Fig 2C).

We further employed an IFN‑β‑GFP reporter system, in which GFP expression is driven by the IFN‑β promoter, to monitor IFN‑β induction. Similar infection assays were performed and analyzed by flow cytometry. Consistent with the above results, viruses produced in NSUN2 KO cells induced markedly higher IFN‑β‑GFP reporter activity compared with those from WT cells, with HSV‑1 infection serving as a positive control (Fig 2D–F). Using a dual‑luciferase reporter assay, we also observed that NSUN2 suppressed poly (I:C)‑induced IFN‑β promoter activation (Fig 2G).

Given that IFN‑I production is a central component of the antiviral innate response during HIV-1 infection, we next investigated whether NSUN2‑mediated enhancement of viral infectivity depends on IFN‑I signaling. To this end, we used WT HeLa cells (IFN‑competent) and IRF3-knockout HeLa cells (IFN‑deficient) as target cells (Fig 2H). Both HeLa cell lines were transfected with VSV-G expression plasmid 24 h before viral infection, followed by incubation with VSV-G pseudotyped HIV-1 virions derived from WT or NSUN2-KO producer cells. While viruses derived from NSUN2-KO and WT producer cells displayed comparable infectivity in IRF3-deficient HeLa cells, their infectivity differed markedly in WT HeLa cells, where virions produced from NSUN2-KO producer cells exhibited reduced infectivity (Fig 2I). These data suggested that NSUN2‑mediated promotion of viral infectivity relies on IFN‑I pathway.

Collectively, these results demonstrate that NSUN2 in viral producer cells attenuates IFN‑β production induced by progeny HIV-1 infection, thereby promoting viral infectivity.

### NSUN2 KO-mediated m5C deficiency in cellular and HIV-1 RNA enhances innate immune response

**Fig 3:**
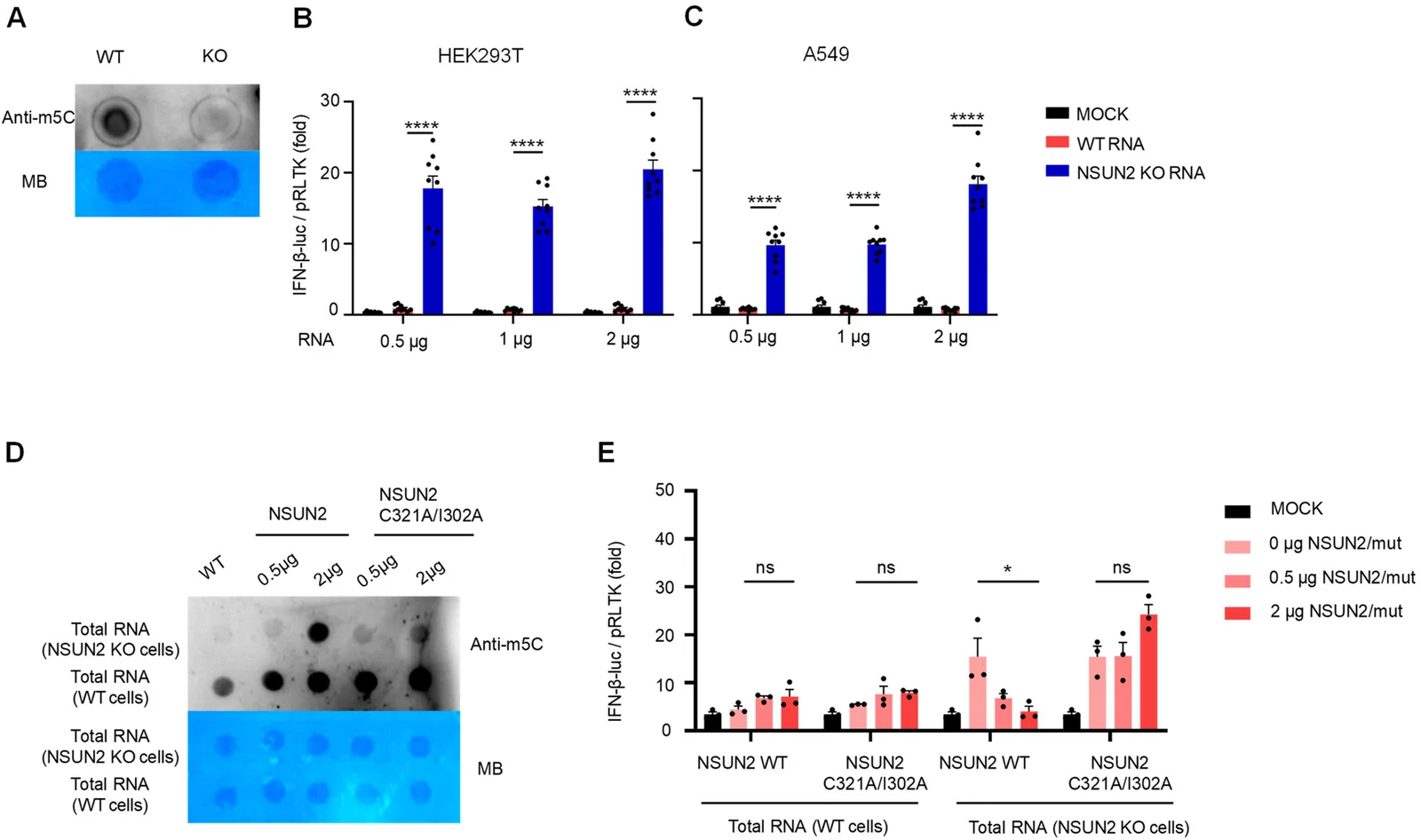
Total RNA from NSUN2 KO cells activates host IFN-β signaling. (A) Representative dot blot analysis of m5C modification in total RNA isolated from WT and NSUN2 KO HEK293T cells (n = 3). (B, C) Dual-luciferase assay measuring IFN-β promoter activity in HEK293T cells or A549 cells stimulated with total RNA as in (A). (D) m5C dot blot analysis of total RNA from WT or NSUN2 KO HEK293T cells reconstituted with WT NSUN2 or catalytically inactive C321A/I302A mutant. (E) Dual-luciferase assay of IFN-β promoter activity in HEK293T cells stimulated with total RNA as in (D). Data are representative of three independent experiments and analyzed by two-tailed unpaired *t*-test or one-way ANOVA. Values are presented as means ± SEM (n = 3). NS, not significant (P > 0.05), *P < 0.05, ****P < 0.0001.

NSUN2 functions as an RNA methyltransferase responsible for m5C deposition on target RNAs. We next sought to determine whether this methyltransferase activity of NSUN2 regulates RNA-triggered IFN‑β induction. To address this question, we utilized total RNA isolated from WT and NSUN2 KO cells to activate the IFN-I response. Total RNA was extracted from WT and NSUN2 KO cells, and dot blot analysis revealed that global m5C modification was dramatically reduced in RNA from NSUN2 KO cells compared with WT cells (Fig 3A). When these RNAs were used to activate RIG-I-like receptor (RLR) signaling pathway, RNA isolated from NSUN2 KO cells with diminished m5C strongly activated IFN‑β production in both HEK293T and A549 cells, whereas RNA from WT cells failed to induce detectable IFN‑β expression (Fig 3B–C). Furthermore, exogenous expression of WT NSUN2 in NSUN2 KO cells restored physiological m5C levels and suppressed RNA‑induced IFN‑β induction, whereas expression of the catalytically inactive NSUN2 C321A/I302A mutant failed to elicit such rescue effects (Fig 3D–E). These results indicate that m5C modification is essential for RNA to evade RLR‑mediated innate immune sensing and that NSUN2 acts as the key methyltransferase responsible for cellular RNA m5C modification.

**Fig 4:**
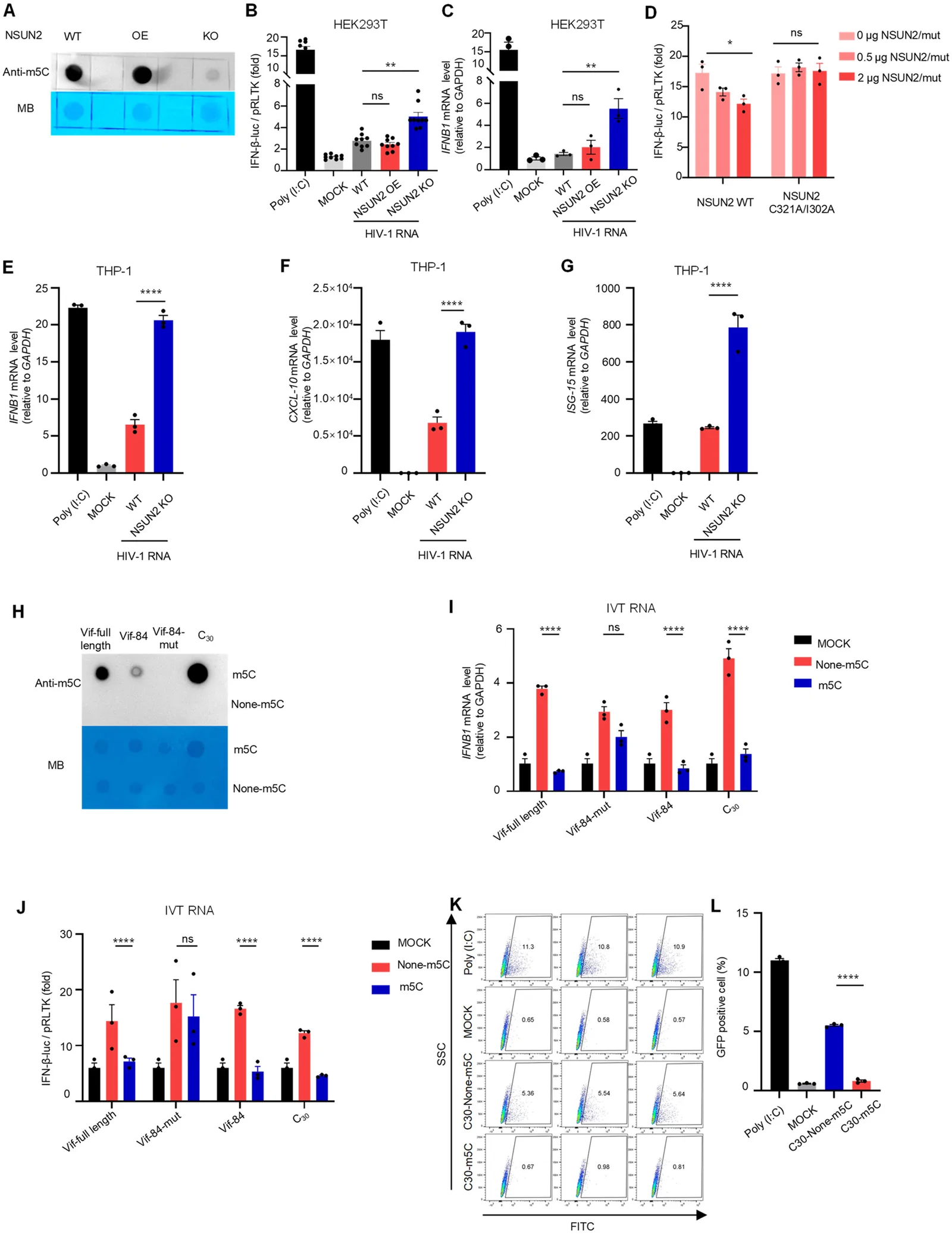
Deficiency of m5C modification enhances HIV-1 RNA-activated IFN-β signaling. (A) m5C dot blot analysis of HIV-1 RNA. The viruses were produced in WT, NSUN2 OE, or NSUN2 KO HEK293T cells. (B) Dual-luciferase assay measuring IFN-β promoter activity in HEK293T cells transfected with viral RNA described in (A), poly (I:C) was used as a positive control. (C) RT-PCR analysis of *IFNB1* mRNA levels in HEK293T cells transfected with viral RNA described in (A). (D) Dual-luciferase assay of IFN-β promoter activity in HEK293T cells activated by viral RNA. NSUN2 KO producer cells were reconstituted with WT NSUN2 or C321A/I302A mutant before generating HIV-1 pseudoviruses. Target HEK293T cells were infected with harvested viruses for luciferase quantification. (E-G) RT-PCR analysis of innate immune-related gene expression in THP-1-derived macrophages. (E) Relative *IFNB1* mRNA levels; (F) Relative *CXCL-10* mRNA levels; (G) Relative *ISG-15* mRNA levels. (H) m5C dot blot analysis of *in vitro* transcribed cap-1 RNAs. IVT RNAs named Vif-full length, vif-84 vif-84-mut, and C30 were transcribed and capped *in vitro* with or without m5C incorporation, followed by dot blot using an anti-m5C antibody. (I) RT-PCR analysis of *IFNB1* mRNA levels in HEK293T cells transfected with IVT RNAs as in (H). (J) Dual-luciferase assay measuring IFN-β promoter activity in HEK293T cells transfected with IVT RNAs as in (H). (K, L) Flow cytometry analysis of the activation of IFN-β-GFP reporter cells induced by the indicated IVT RNAs. Data are representative of three independent experiments and analyzed by two-tailed unpaired *t*-test or one-way ANOVA. Values are presented as means ± SEM (n = 3). NS, not significant (P > 0.05), **P < 0.01, ***P < 0.001, ****P < 0.0001.

Given that HIV-1 genomic RNA has been reported to carry m5C modification, we hypothesized that NSUN2‑mediated m5C methylation of viral RNA shields the HIV-1 genome from detection by the host innate immune system, thereby enabling immune evasion during viral infection. To test this hypothesis, we produced HIV-1 viral particles in WT, NSUN2‑overexpressing, and NSUN2 KO producer cells, extracted viral RNA from purified virions and evaluated their ability to activate the IFN-I pathway. Dot blot analysis confirmed that m5C modification was markedly reduced in viral RNA derived from NSUN2 KO producer cells (Fig 4A), and correspondingly, viral RNA lacking m5C induced significantly higher levels of IFN‑β as measured by both dual‑luciferase reporter assay and RT‑PCR (Fig 4B–C). Additionally, we performed rescue assays with WT NSUN2 and its catalytically dead C321A/I302A mutant to test if the catalytic activity of NSUN2 drives the observed phenotype via modification of viral RNA. Briefly, NSUN2-KO producer cells were transfected with WT NSUN2 or C321A/I302A mutant prior to generating VSV-G pseudotyped HIV-1 virions. We then infected target cells with these viruses and measured innate immune responses. Consistent with our earlier observations regarding cellular RNAs, NSUN2 C321A/I302A mutant failed to elicit rescue effects (Fig 4D). These results confirm that the immune evasion phenotype relies specifically on the catalytic activity of NSUN2 toward viral RNA.

To further validate these findings in physiologically relevant HIV-1 target cells, we repeated the immune stimulation assay using THP-1-derived macrophages. Briefly, THP-1 monocytes were differentiated into macrophages via PMA treatment, then transfected with the above purified viral genomic RNA, with poly (I:C) serving as a positive control. Total cellular RNA was collected 24 h post-transfection to measure *IFNB1*, *CXCL-10* and *ISG-15* mRNA level. Consistently, viral RNA extracted from NSUN2-KO-derived virions triggered markedly stronger innate immune responses (Fig 4E–G). Together, these results demonstrate that NSUN2-dependent m5C modification of viral RNA promotes HIV-1 immune evasion across multiple cellular systems.

A previous study identified a functional m5C site within the overlapping region between the carboxyl terminus of Pol and the amino terminus of *vif* [32]. Considering our above findings that NSUN2-mediated m5C modification suppresses HIV-1-triggered IFN-I activation, we therefore sought to determine whether m5C modification in this region contributes to the regulation of IFN-I induction. To address this, we *in vitro*-transcribed full-length vif RNA, an 84-nucleotide fragment containing the m5C site (vif-84), a cytosine-mutated version of vif-84 (vif-84-mut), and a 30-cytidine RNA (C_30_) in the presence or absence of m5C incorporation. Dot blot assay confirmed efficient m5C incorporation in full-length vif, vif-84, and C_30_ transcripts (Fig 4H). Compared with unmodified RNAs, these m5C-modified RNAs elicited significantly lower IFN-β induction (Fig 4I–J). As expected, m5C incorporation had no effect on vif‑84‑mut RNA due to the absence of target cytosines. Similar results were obtained using an IFN‑GFP reporter cell line, confirming that m5C‑modified RNAs were nearly unable to activate IFN-I signaling (Fig 4K–L). Consistent results obtained from two separate experimental assays strengthen the reliability of our findings, affirming that the m5C sites of Pol-vif overlapping region serve as the functional motif mediating NSUN2-dependent innate immune evasion during HIV-1 infection.

Collectively, these data demonstrate that m5C modification allows both cellular and viral RNA to evade the IFN-I‑mediated innate immune response.

### Loss of m5C in HIV NL4–3 laboratory strain RNA enhances the RNA-mediated innate immune response

**Fig 5:**
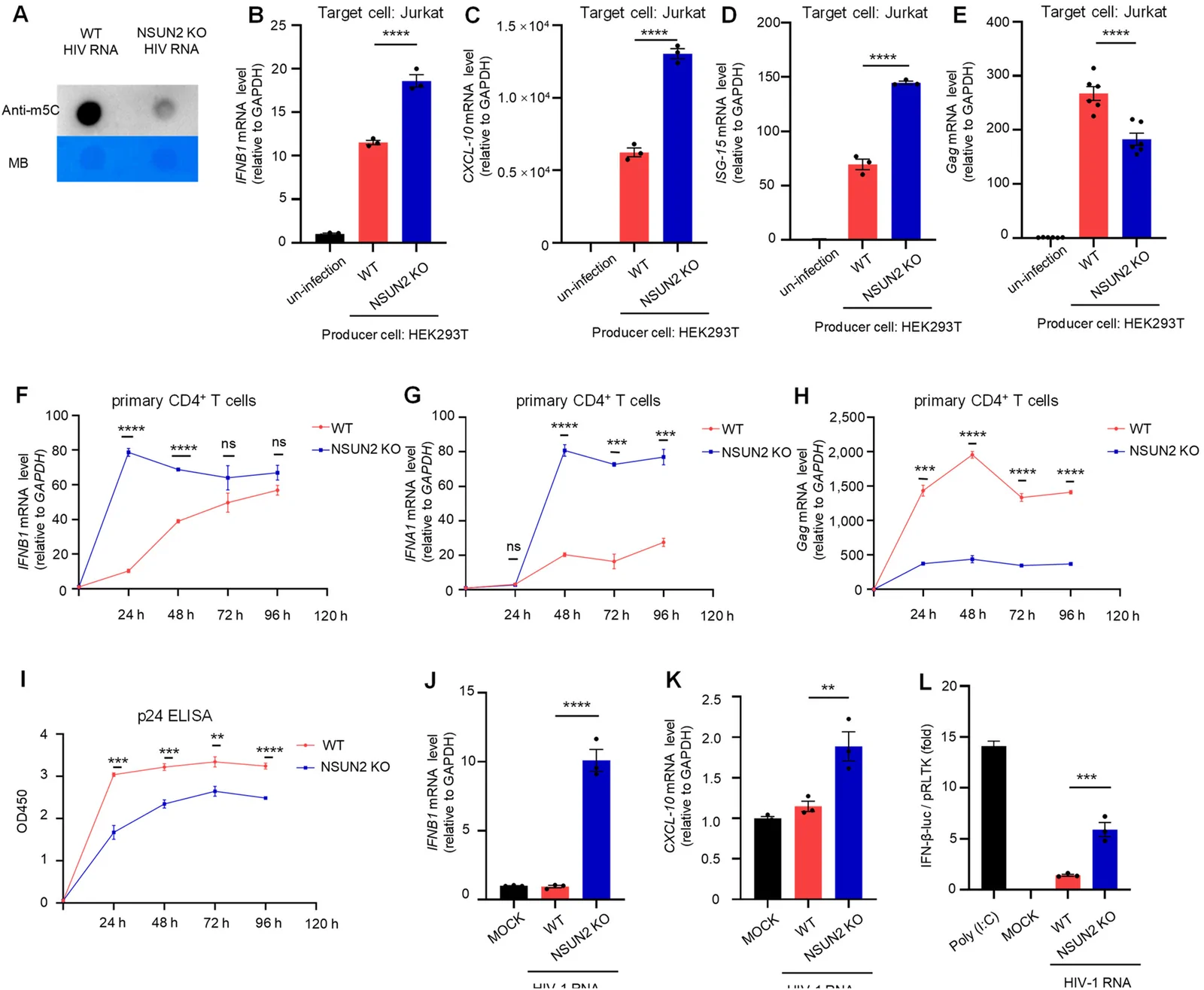
Loss of m5C modification in authentic HIV‑1 RNA enhances RNA‑mediated innate immune responses. (A) m5C dot blot analysis of genomic HIV‑1 RNA isolated from viruses produced in WT or NSUN2 KO cells. (B-E) RT-PCR analysis of *IFNB1*, *CXCL-10*, *ISG-15*, and Gag mRNA levels in Jurkat cells infected with HIV-1 derived from WT or NSUN2 KO producer cells. (F-I) Innate immune response and viral replication dynamics in primary human CD4 ⁺ T cells infected with WT or NSUN2-KO NL4–3 HIV-1 at 24, 48, 72 and 96 h post-infection. (F) Relative *IFNB1* mRNA levels detected by RT-PCR; (G) Relative *IFNA1* mRNA levels; (H) Gag mRNA level; (I) Viral particle release in culture supernatants quantified by p24 ELISA. (J-K) RT-PCR analysis of *IFNB1* and *CXCL-10* mRNA levels in HEK293T cells transfected with HIV-1 RNA described in (A). (L) Dual-luciferase assay measuring IFN-β promoter activity in HEK293T cells transfected with HIV-1 RNA described in (A). Data are representative of three independent experiments and analyzed by two-tailed unpaired *t*-test or one-way ANOVA. Values are presented as means ± SEM (n = 3). **P < 0.01, ***P < 0.001, ****P < 0.0001.

Having established that m5C-modified viral RNA is less immunostimulatory in reductionist assays, we next tested whether the same principle applies to authentic replicating HIV-1. To further examine whether viral RNA m5C modification is critical for IFN induction mediated by authentic HIV-1 genomic RNA, we employed the laboratory HIV strain NL4‑3. NL4‑3 viruses were produced in WT HEK293T cells or NSUN2 KO cells and harvested for subsequent analysis. Dot blot analysis revealed that viral genomic RNA derived from NSUN2 KO cells exhibited a marked loss of m5C modification (Fig 5A). These viruses were then used to infect Jurkat cells, and total RNA was extracted for RT‑PCR analysis. Compared with infection by HIV-1 carrying m5C‑modified viral RNA, infection by HIV-1 lacking m5C modification resulted in significantly increased mRNA levels of *IFNB1*, *CXCL‑10*, and *ISG‑15* (Fig 5B–D), whereas Gag mRNA levels were reduced (Fig 5E). To validate these observations in physiologically more relevant primary cells, we performed similar experiments in primary human CD4 ⁺ T cells. We infected primary human CD4 ⁺ T cells with authentic HIV-1 particles generated from WT or NSUN2-KO producer cells and collected cell pellets and culture supernatants at serial time points of 24, 48, 72, and 96 h post-infection. Cellular RNA was extracted at indicated time points for RT-qPCR quantification of *IFNB1* (Fig 5F), *IFNA1* (Fig 5G) and viral Gag mRNA (Fig 5H). Meanwhile, viral production in culture supernatants was determined via p24 ELISA (Fig 5I). Consistent with the results obtained in Jurkat cells, loss of NSUN2-dependent m5C methylation on viral RNA markedly enhanced cellular innate immune activation and significantly suppressed viral replication in primary human CD4 ⁺ T cells. Results from live HIV-1 infection assays using primary CD4 ⁺ T cells can partially recapitulate the physiological immune evasion driven by viral m5C modification during HIV-1 infection.

Innate immune activation induced by viral infection is susceptible to various confounding variables, including viral entry efficiency and viral or host proteins that regulate immune signaling pathways. We thus sought to confirm that the phenotype detected in our study is directly mediated by HIV-1 RNA m5C modification. To directly verify that viral RNA m5C modification attenuates RNA‑triggered immune activation, viral RNA was extracted from NL4–3 virions produced in WT and NSUN2 KO cells and transfected into HEK293T cells. RT‑PCR demonstrated that viral RNA lacking m5C strongly activated IFN-I signaling, whereas WT m5C‑modified viral RNA did not (Fig 5J–K). Dual‑luciferase reporter assays further confirmed that m5C‑deficient viral RNA potently induced IFN-I production, with poly (I:C) used as a positive control (Fig 5L). This transfection experiment provides direct evidence that differing m5C modification status of purified viral RNA alone accounts for differential innate immune activation, independent of other infection-related cellular or viral factors.

Collectively, these data demonstrate that NSUN2 serves as a key methyltransferase responsible for m5C modification of authentic HIV-1 genomic RNA, and that such viral RNA methylation enables HIV-1 to evade detection by the host innate immune system.

### RIG-I is the sensor for non-m5C RNA

**Fig 6:**
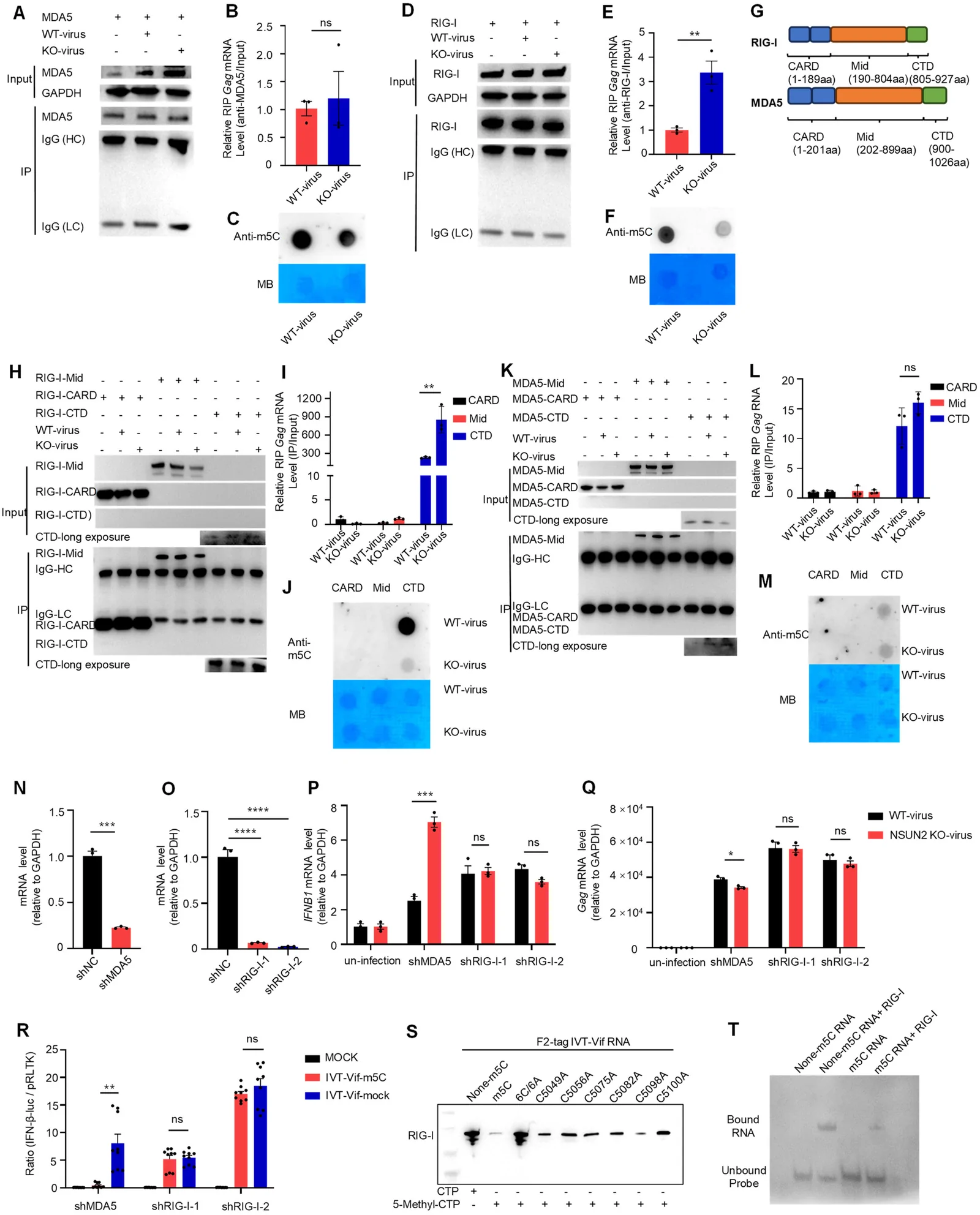
The m5C modified RNA evades innate immunity by reducing binding to RIG-I. (A-C) RIP assay detecting viral RNA co-precipitated with MDA5 (n = 3). HEK293T cells expressing FLAG‑tagged MDA5 were infected with pseudotyped HIV-1 produced in WT or NSUN2 KO cells. At 2 h post‑infection, cell lysates were subjected to immunoprecipitation with anti‑FLAG beads (A). Co‑precipitated RNA was analyzed by RT‑PCR (B) and m5C dot blot (C). (D-F) RIP assay detecting viral RNA co-precipitated with RIG-I (n = 3). HEK293T cells expressing FLAG‑tagged RIG-I were infected with pseudotyped HIV-1 produced in WT or NSUN2 KO cells. At 2 h post‑infection, cell lysates were subjected to immunoprecipitation with anti‑FLAG beads (D). Co‑precipitated RNA was analyzed by RT‑PCR (E) and m5C dot blot (F). (G) Schematic diagram of RIG-I and MDA5 truncations. (H-J) RIP assay detecting viral RNA co-precipitated with the indicated truncated RIG-I. (K-M) RIP assay detecting viral RNA co-precipitated with the indicated truncated MDA5. (N-O) Knockdown efficiency of MDA5 and RIG-I in shRNA-mediated stable knockdown cell lines. (P-Q) RT-PCR analysis of *IFNB1* and Gag mRNA levels in MDA5 and RIG-I knockdown cell lines infected with HIV-1 derived from WT or NSUN2 KO cells. (R) Dual-luciferase assay measuring IFN-β promoter activity in MDA5 and RIG-I knockdown cell lines transfected with IVT RNAs. (S) RNA pull-down analysis of the interaction between RIG-I and different vif RNA. F2-tagged in vitro transcribed vif RNAs (with or without m5C modification) were incubated with cell lysates overexpressing RIG-I. RNA-protein complexes were enriched and subjected to immunoblotting to detect bound RIG-I protein. (T) Electrophoretic mobility shift assay (EMSA) for detecting the binding of recombinant RIG-I to 5′ biotin-labeled RNA with or without m5C modification. Mixtures of RIG-I protein and RNA probes were separated by native gel electrophoresis to separate free RNA and RNA-protein complexes. Data are representative of three independent experiments and analyzed by two-tailed unpaired *t*-test or one-way ANOVA. Values are presented as means ± SEM (n = 3). **P < 0.01, ***P < 0.001, ****P < 0.0001.

Given that RIG‑I and MDA5 are the two major cytoplasmic RNA sensors both reported to recognize HIV-1 RNA [37–39], we sought to determine which sensor distinguishes between m5C‑modified and unmodified viral RNA. To address this, we overexpressed FLAG‑tagged MDA5 or RIG‑I in HEK293T cells, followed by infection with pseudotyped HIV-1 produced in either WT or NSUN2 KO cells. At 2 hours post‑infection, cells were lysed and FLAG‑tagged proteins were immunoprecipitated using anti‑FLAG beads. Co‑precipitated RNA was analyzed by RT‑PCR and dot blot. The results showed that non‑m5C viral RNA was not enriched in MDA5 immunoprecipitates (Fig 6A–C). Notably, non‑m5C viral RNA was markedly enriched in RIG‑I immunoprecipitates compared with m5C‑modified viral RNA, with comparable levels of RIG‑I protein pulled down (Fig 6D–E). Dot blot analysis further confirmed the m5C modification status of viral RNA co‑precipitated with RIG‑I (Fig 6F). These data indicate that RIG‑I, but not MDA5, preferentially binds non‑m5C viral RNA.

To identify which domain mediates the recognition of non‑m5C RNA, we truncated RIG‑I and MDA5 into three segments: the CARD domain, the middle region (Mid), and the C‑terminal domain (CTD) (Fig 6G). RNA immunoprecipitation (RIP) assays were performed using these truncated constructs during infection with HIV-1 produced from WT or NSUN2 KO cells. Western blot analysis confirmed efficient expression and immunoprecipitation of each domain (Fig 6H and 6K). Strikingly, non‑m5C viral RNA was significantly enriched by the RIG‑I CTD domain (Fig 6I). Although the CTD domains of both RIG‑I and MDA5 bound viral RNA, MDA5 exhibited a much lower binding capacity (Fig 6I and 6L). The m5C modification status of co‑precipitated RNA was verified by dot blot (Fig 6J and 6M).

To further confirm the role of RIG‑I in sensing non‑m5C RNA, we established RIG‑I or MDA5 knockdown cell lines using shRNAs. Knockdown efficiency was validated as shown in Fig 6N–O. Two shRNAs were designed for each target, but only one shRNA against MDA5 achieved efficient knockdown. These cell lines were then infected with HIV-1 produced from WT or NSUN2 KO cells, and immune activation was measured by RT‑PCR. Compared with viruses from WT cells, viruses derived from NSUN2 KO cells still induced higher *IFNB1* mRNA expression in MDA5‑knockdown cells, whereas no such difference was observed in RIG‑I‑knockdown cells (Fig 6P). Consistent with this, viral replication was moderately decreased in MDA5‑knockdown cells (Fig 6Q). Moreover, *in vitro*‑transcribed non‑m5C RNA elicited significantly higher IFN‑β induction than m5C‑modified RNA in MDA5‑knockdown cells, whereas no difference between non‑m5C and m5C RNA was observed in RIG‑I‑knockdown cells (Fig 6R).

To further validate the physical interaction between RIG‑I and unmethylated viral RNA, we conducted RNA pull‑down assays and electrophoretic mobility shift assays (EMSA). For RNA pull-down assay, F2-tagged in vitro transcribed vif RNAs (including unmodified full-length, m5C-modified full-length, and 6C-to-6A as well as single-point mutants C5049A, C5056A, C5075A, C5082A, C5098A and C5100A) with or without m5C modification were incubated with lysates from RIG-I-overexpressing cells. Captured RNA-protein complexes were subjected to immunoblotting to detect bound RIG-I protein. Data revealed that m5C-modified RNA exhibited markedly weaker binding capacity to RIG-I than unmodified RNA (Fig 6S, Lanes 1 and 2). Moreover, single-point mutations at six cytosine residues enhanced RNA-RIG-I binding relative to unmutated m5C-containing vif RNA, whereas the C5098A mutation only induced mild enhancement of this interaction (Fig 6S). For EMSA assays, recombinant RIG-I protein was incubated with 5′ biotin-labeled RNA substrates bearing or lacking m5C modification, followed by native gel electrophoresis to separate free RNA and RIG-I–RNA complexes. Consistent with RNA pull-down results, shifted bands corresponding to RIG-I-RNA complexes were far less abundant for m5C-modified RNA compared with unmodified RNA (Fig 6T, Lanes 2 and 4). Collectively, RNA pull-down and EMSA experiments consistently confirm that m5C modification impairs the direct association between vif RNA and RIG-I, and RNA pull-down further reveals that mutating specific cytosine residues can reverse this inhibitory binding effect.

Taken together, these data demonstrate that RIG‑I, rather than MDA5, acts as the primary sensor for non‑m5C viral RNA during HIV-1 infection, and that the CTD domain of RIG‑I is responsible for binding with non‑m5C RNA.

## Discussion

**Fig 7:**
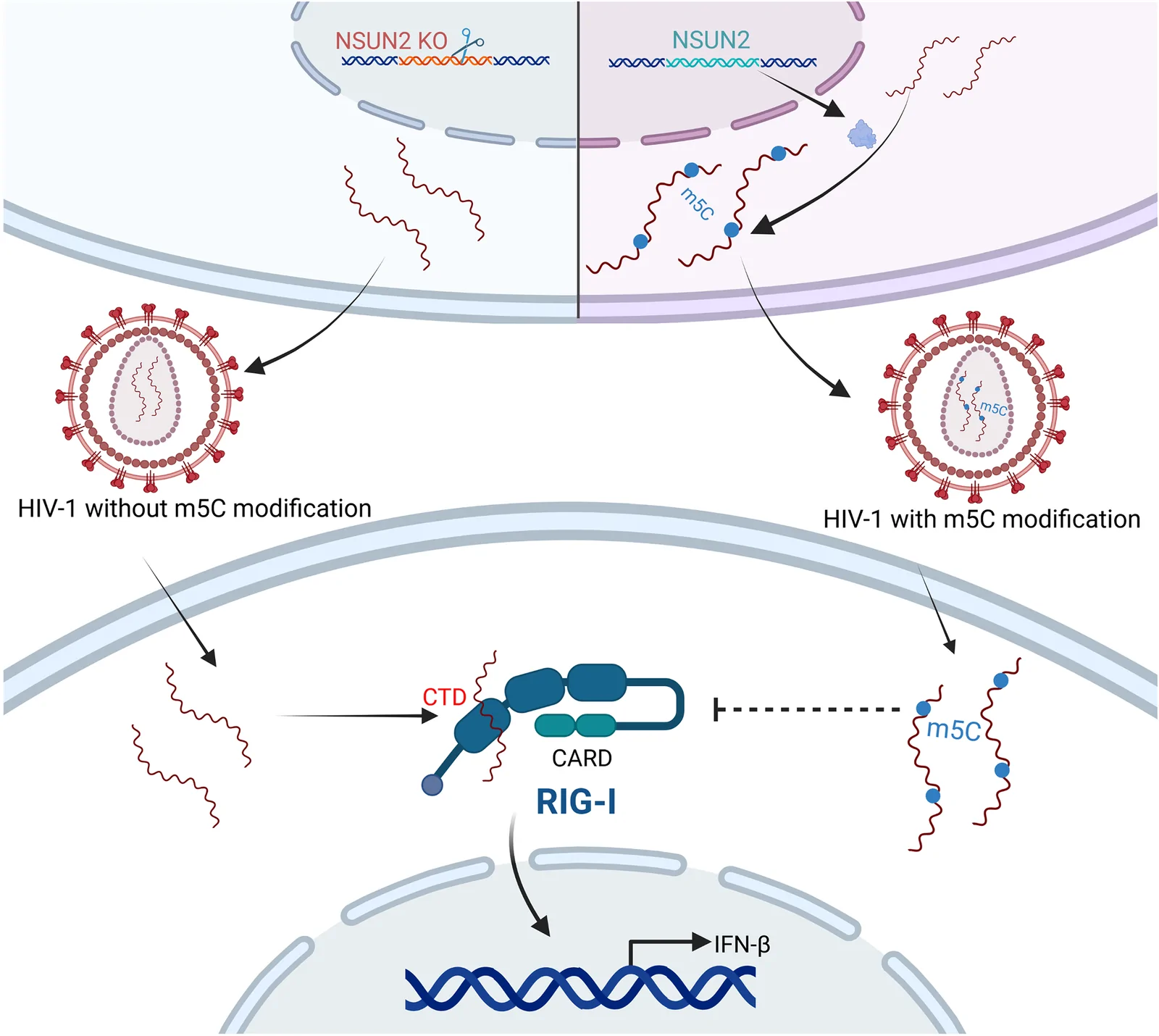
Schematic model of NSUN2-mediated m5C modification on HIV-1 RNA enabling evasion of RIG‑I‑dependent innate immunity. During HIV-1 production, host cellular NSUN2 mediates m5C modification of HIV‑1 genomic RNA. m5C-modified HIV‑1 RNA exhibits reduced binding affinity to the C-terminal domain (CTD) of RIG-I, thereby allowing HIV-1 to evade host innate immune surveillance. In contrast, HIV‑1 RNA lacking m5C modification efficiently interacts with RIG‑I, triggering downstream signaling and activating the IFN‑β signaling pathway (Created in BioRender. https://BioRender.com/b0jd9zj).

Our studies reveal a novel role of m5C modification in HIV-1 viral RNA to evade host innate immunity that supports efficient viral replication. A schematic model summarizing our findings is presented in Fig 7 Progeny HIV-1 from WT cells harbor m5C modification on their viral RNA; upon infecting target cells, these modifications prevent viral RNA from being recognized by RIG-I and enable evasion of the innate immune response. In contrast, progeny HIV-1 produced from NSUN2 KO cells lack sufficient m5C modification on their viral RNA, leading to the RNA being detectable by RIG-I and triggering IFN-I production upon viral infection, thereby potently inhibiting viral replication. This finding uncovers a previously uncharacterized mechanism by which HIV-1 hijacks the host RNA modification machinery to modify its own RNA, thus masking itself from host immune detection and achieving successful infection.

The link between NSUN2-mediated m5C modification and the regulation of innate immune responses has been previously established in a cellular context by Zhang and colleagues, who reported that m5C RNA modification controls antiviral innate immunity by modulating the m5C methylome of non-coding RNAs (ncRNAs) and their expression [33]. In their study, NSUN2 depletion led to the upregulation of cytoplasmic 7SL RNA, which serves as a direct ligand for RIG-I and triggers RIG-I-mediated IFN-I production, which was highly consistent with our findings. In the present work, we show that total cellular RNA from NSUN2 KO cells potently activates host IFN-I production (Fig 3), and this immune activation can be fully rescued by the overexpression of WT NSUN2 but not the catalytically inactive NSUN2 C321A/I302A mutant. This result demonstrates that loss of m5C modification causes cellular RNA to become immunostimulatory, independently of off-target effects caused by NSUN2 depletion. It validates the general principle that unmodified cytosines in RNA act as a molecular pattern recognized by the innate immune system [40]. Extending this principle to viral RNA, our data further reveal that HIV-1 utilizes this host epigenetic pathway: by acquiring NSUN2-mediated m5C modification, HIV-1 RNA evades the same RIG-I-dependent sensing mechanism, thereby suppressing the innate immune response and enabling efficient viral replication.

Our functional validation in IFN-deficient cells and IFN-competent cells further supports the causal link between m5C-mediated immune evasion and increased HIV-1 infectivity (Fig 2I). Moreover, experiments using the authentic HIV NL4–3 strain confirm the physiological relevance of our results: m5C-deficient viral RNA strongly induces *IFNB1*, *CXCL-10*, and *ISG-15* expression and decreases Gag mRNA levels (Fig 5). These data confirm that the m5C-RIG-I-IFN axis is a critical regulator of authentic HIV-1 infection, not just pseudotyped virus models.

A key finding of our study is the identification of RIG-I, but not MDA5, as the primary innate immune sensor that discriminates between m5C-modified and unmodified HIV-1 viral RNA, which was consistent with previous studies [31,33]. Our domain mapping studies further identify the C-terminal domain (CTD) of RIG-I as the critical region mediating the binding to non-m5C HIV-1 RNA, while the CTD of MDA5 exhibits only weak binding capacity for viral RNA regardless of m5C status (Fig 6). The selective recognition of unmodified HIV-1 RNA by RIG-I suggests that m5C modification may alter the structural features of HIV-1 RNA or directly block the RNA-binding interface of RIG-I, which remains to be elucidated by structural studies. Notably, this mechanism is similar to the immune evasion strategy used by HBV, in which viral RNA m5C modification inhibits RIG-I binding and IFN-β production [31]. This indicates a conserved role of viral RNA m5C modification in evading RIG-I-mediated innate immunity among different viruses.

While our work establishes a critical role for NSUN2-mediated m5C modification in HIV-1 immune evasion, several questions remain to be addressed. First, the precise m5C modification sites on the HIV-1 genome that mediate RIG-I evasion require further mapping. Second, the molecular mechanism by which m5C modification blocks RIG-I binding remains unclear and requires further structural analysis to determine whether m5C alters RNA secondary structure or directly interacts with the RIG-I CTD. Third, it remains unclear whether m5C reader proteins, such as ALYREF, contribute to HIV-1 immune evasion by regulating the recognition of unmodified viral RNA by RIG‑I. Finally, the clinical relevance of HIV-1 RNA m5C modification remains to be explored, and whether the m5C modification level correlates with viral load, disease progression, or antiretroviral therapy response in HIV-infected patients is an important area for future clinical research.

A key limitation of this study resides in the broad substrate range of NSUN2. Apart from HIV-1 RNA, NSUN2 introduces m5C modifications to numerous cellular tRNAs, non-coding RNAs, and mRNAs [41,42]. NSUN2 knockout thus causes global alterations in cellular RNA stability, translation and gene expression, which may indirectly interfere with immune signaling and viral replication. While our viral RNA transfection assays in WT cells confirm that m5C-deficient viral RNA alone drives enhanced IFN responses, we cannot fully exclude indirect effects derived from disrupted host RNA methylation, since HIV-1 virions naturally package host tRNAs to serve as primers for reverse transcription [43,44], and these co-packaged cellular tRNAs may exhibit disrupted m5C modifications upon NSUN2 deletion. Future studies using viral mutants deficient in viral m5C sites will better distinguish viral-specific phenotypes from cellular off-target effects. An additional limitation is that the viral m5C modification sites used in our research were obtained from previous reports [32], and high-precision m5C sequencing was not performed to independently verify these loci. Nevertheless, we validated the relevant phenotypes via site-directed mutagenesis, particularly regarding the RNA binding affinity to RIG-I and its capacity to trigger innate immune activation.

In summary, our study identifies a novel epigenetic immune evasion mechanism of HIV, wherein NSUN2-mediated m5C modification of viral RNA prevents RIG-I recognition, suppresses IFN-I production, and thereby promotes efficient viral replication. This finding not only expands our understanding of the complex interplay between HIV-1 and the host innate immune system, but also uncovers a conserved role for viral RNA m5C modification in evading RIG-I-mediated sensing across diverse viruses. Furthermore, our work highlights NSUN2 and the m5C modification machinery as potential novel therapeutic targets for HIV-1 infection. Strategies that inhibit NSUN2 activity or reduce HIV-1 RNA m5C modification could restore RIG-I-mediated immune recognition of HIV-1, enhance the innate antiviral response, and improve viral clearance.

## Materials and methods

### Ethics statement

Human PBMCs were purchased from MILECELL BIO, and all samples were fully anonymized. The ethics committee of the School of Basic Medical Sciences, Fudan University has approved the in vitro experiments described in this study, as no identifiable human information was used throughout all experimental procedures.

### Cell culture

HEK293T (CRL-3216), THP-1 (TIB-202), A549 (CCL-185), and Jurkat (TIB-152) cells were obtained from MeilunBio. HeLa (CCL-2) cells were obtained from Fuheng Biology. HeLa IRF3-KO cell line was kindly provided by Prof. Gang Long (Fudan University). HEK293T NSUN2 KO, shMDA5, shRIG-I, HeLa NSUN2 KO, and HeLa-IFN-β-GFP cell lines were generated in our laboratory. The guide RNA (gRNA) sequence for NSUN2 KO is 5’-GACGCGGAGGATGGCGCCGA-3’. The shRNA sequence for MDA5 is 5’-GCATCTCTTCAATACCCTTCA-3’. The shRNA sequences for RIG-I are 5’-AGCACTTGTGGACGCTTTAAA-3’ and 5’-CCAGAATTATCCCAACCGATA-3’. The stable cell lines expressing target shRNA were established as previously described [45]. HEK293T, A549 and HeLa cells were maintained in Dulbecco’s modified Eagle’s medium (DMEM), while Jurkat cells and THP-1 cells were maintained in RPMI 1640 medium. All media were supplemented with 10% fetal bovine serum, 100 U/mL penicillin and 100 μg/mL streptomycin. Cells were cultured at 37℃ in a humidified incubator with 5% CO_2_.

### Viruses and reagent

HIV-1 pseudoviruses were generated by co-transfecting HEK293T or HeLa cells with plasmids VSVG and pNL4–3-R ⁺ E^−^ at a ratio of 1:3. m5C modification-deficient HIV-1 pseudoviruses were packaged in the corresponding NSUN2 KO cell lines using the same plasmid ratio. All HIV-1 pseudovirus stocks were normalized according to p24 protein level prior to infection of target cells for infectivity quantification. Infected cells were harvested and lysed at 36 h post-infection for firefly luciferase activity measurement. Raw Relative Light Unit (RLU) values reflecting HIV infectivity were first normalized to sample total protein, and those normalized values were compared between groups to obtain relative infectivity differences in viral infectivity. Authentic HIV-1 was produced by transfecting pNL4–3 into HEK293T or HEK293T-NSUN2 KO cells. Herpes simplex virus (HSV-1) was kindly provided by Dr. Dapeng Yan.

HIV-1 p24 antibody (11695-R002) was purchased from SinoBiological. GAPDH antibody (0411) (sc-47724) was purchased from Santa Cruz Biotechnology. HA tag Polyclonal antibody(51064–2-AP), NSUN2 Polyclonal antibody(20854–1-AP) and HRP-Goat Anti-Mouse Recombinant Secondary Antibody (RGAM001) were purchased from Proteintech. 5-methylcytosine antibody (ab10805) was purchased from Abcam. Goat Anti-Rabbit IgG (whole molecule)-HRP (A6154) was purchased from sigma. Actinomycin D (A113142) and Methylene Blue (M196501) were purchased from Aladdin. Lipofectamine RNAiMAX (13778150), Lipofectamine 2000 (11668019) and TRIzol (15596018CN) were purchased from Invitrogen.

### HIV-1 virion purification

Briefly, culture supernatants were collected from pNL4–3 transfected HEK293T or HEK293T NSUN2 KO cells at 48 h and 72 h post-transfection, and filtered through a 0.45 μm membrane to remove cell debris. Virions were then purified by ultracentrifugation at 100,000 × g for 90 min at 4 ℃, using a 20% (w/v) sucrose cushion.

### RNA isolation and quantitative real-time PCR

Total RNA was isolated from cells using TRIzol reagent according to the manufacturer’s instructions. cDNA was synthesized from 1 μg of total RNA using the Hifair Ⅱ 1st Strand cDNA Synthesis Kit (11121ES60, YEASEN) following the kit protocol. Real-time PCR was performed with Hieff qPCR SYBR Green Master Mix (11203ES08, YEASEN) on QuantStudio 5 (Thermofisher). The relative expression levels of target genes were calculated using the 2^−^ΔΔCt method and normalized to GAPDH as an internal control. Primer sequences are shown below:

Gag-F: GTGTGGAAAATCTCTAGCAGTGG

Gag-R: CGCTCTCGCACCCATCTC

Tat-Rev-F: ATGGCAGGAAGAAGCGGAG

Tat-Rev-R: ATTCCTTCGGGCCTGTCG

CXCL10-F: GGAACCTCCAGTCTCAGCACCA

CXCL10-R: AGACATCTCTTCTCACCCTTC

IFNB1-F: AACTTTGACATCCCTGAGGAGATTAAGC

IFNB1-R: GACTATGGTCCAGGCACAGTGACTGTAC

GAPDH-F: GGAAGGTGAAGGTCGGAGTCAACGG

GAPDH-R: CTGTTGTCATACTTCTCATGGTTCAC

IL-6-F: ACTCACCTCTTCAGAACGAATTG

IL-6-R: CCATCTTTGGAAGGTTCAGGTTG

### Western blotting

Cells were harvested and lysed in RIPA buffer (50 mM Tris pH7.5, 150 mM NaCl, 1 mM EDTA, 1% TritonX-100, 0.1% SDS, and protease inhibitor cocktail). Cell lysates were clarified by centrifugation at 12,000 × g for 10 min at 4 ℃. Protein samples were mixed with SDS loading buffer and boiled at 100 ℃ for 10 min. Equal amounts of protein were separated by 10% SDS-PAGE, transferred onto nitrocellulose (NC) membranes, and blocked with 5% milk in TBST for 1 h at room temperature. Membranes were then incubated with primary antibodies overnight at 4 ℃, followed by incubation with HRP-conjugated secondary antibodies for 1 h at room temperature. Protein bands were visualized using an ECL detection system. Band gray values were quantified using ImageJ software. Target protein expression levels were normalized to internal reference proteins. All Western blotting experiments were conducted with three independent biological replicates (n = 3).

### m5C dot blot assay

Equal amounts of RNA (400 ng) were denatured at 65℃ for 10 min, followed by immediate chilling on ice. Denatured RNA was then loaded onto a nitrocellulose membrane. After UV crosslinking at 254 nm for 30 min, the membrane was blocked with 5% non-fat dry milk in PBS for 1 h. The membrane was incubated with primary anti-m5C antibody (1:500 dilution) overnight at 4℃, washed three times with PBST for 5 min each, and then incubated with goat anti-mouse IgG-HRP for 1h at room temperature. After another three washes, the m5C RNA levels were visualized by ECL detection system. Equal RNA loading was verified by methylene blue (MB) staining. All dot blot assays were conducted with three independent biological replicates (n = 3).

### *In vitro* transcription and capping of RNA

*In vitro* transcription to generate cap1-modified RNA was performed using the T7 High Yield RNA Synthesis Kit for Co-transcription (10673ES60, YEASEN) following the manufacturer’s instructions. Briefly, linearized DNA templates with T7 promoter sequences were used to assemble the 20 μL transcription reaction system (1x Transcription Buffer, 10 mM each of ATP, CTP, GTP and N¹-Me-Pseudo UTP, 10 mM GAG, 1 μg template DNA, 2 μL T7 RNA Polymerase Mix). The reaction was incubated at 37 ℃ for 3 h, followed by DNase I treatment (37 ℃, 15 min) to remove template DNA. For m5C-modified RNA preparation, CTP was replaced with 5-Methylcytidine-5’-triphosphate Sodium Salt (TCI, M3583) at the same concentration. RNA was purified by lithium chloride precipitation, washed with 70% ice-cold ethanol, and dissolved in RNase-free water. The concentration and integrity of the synthesized cap1 RNA were verified by UV absorption and agarose gel electrophoresis. Sequences are shown below:

C_30_-F: 5’-TAATACGACTCACTATAAGGCCCCCCCCCCCCCCCCCCCCCCCCCCCCCC-3’

C_30_-R: 5’-GGGGGGGGGGGGGGGGGGGGGGGGGGGGGGCCTTATAGTGAGTCGTATTA-3’

Vif 84-mut-F: 5’-TAATACGACTCACTATAAGGGATTATGGAAAAAAGATGGAAGGTGATGATTGTGTGGAAAGTAGAAAGGATGAGGATTAATATA-3’

Vif 84-mut-R: 5’-TATATTAATCCTCATCCTTTCTACTTTCCACACAATCATCACCTTCCATCTTTTTTCCATAATCCCTTATAGTGAGTCGTATTA-3’

Vif 84 -F: 5’-TAATACGACTCACTATAAGGGATTATGGAAAACAGATGGCAGGTGATGATTGTGTGGCAAGTAGACAGGA TGAGGATTAACACA-3’

Vif 84-R: 5’-TGTGTTAATCCTCATCCTGTCTACTTGCCACACAATCATCACCTGCCATCTGTTTTCCATAATCCCTTATAGT GAGTCGTATTA-3’

### Flow cytometry

For HIV-1-GFP infectivity detection or IFN-β-GFP reporter assay, target cells were harvested at indicated time points and washed twice with cold PBS/2% FBS to remove residual culture medium. Cells were fixed with 4% paraformaldehyde for 10 min at room temperature, followed by another two washes with cold PBS/2% FBS. After fixation, cells were resuspended in PBS. Cellular GFP fluorescence signals were directly acquired by flow cytometer, and untreated cells were used to set the fluorescence threshold for background normalization. The proportion of GFP-positive cells and mean fluorescence intensity (MFI) were analyzed using FlowJo software.

### Dual-Luciferase reporter assay

Cells were seeded into 24-well plates and co-transfected with 200 ng of the luciferase reporter plasmid, NSUN2 or empty control plasmid, and 20 ng of pRL-TK Renilla luciferase plasmid (for normalization). Poly (I:C) or RNAs were transfected into cells by Lipofectamine RNAiMAX reagent. Luciferase activity in cell lysates was measured at 36 h post-transfection by using a dual-luciferase reporter assay system (E1960, Promega).

### RNA immunoprecipitation (RIP) assay

HEK293T cells were seeded at 2 million cells per 6 cm dish and transfected with 3 μg Flag-tagged target protein plasmids on the next day. Twelve hours post-transfection, cells were incubated with equal-titer pseudoviruses generated from WT or NSUN2-KO HEK293T cells for 2 h. Cells were washed with PBS, harvested, and lysed in RIPA buffer supplemented with protease inhibitors on ice for 40 min. After centrifugation to remove cell debris, clarified lysates were divided into three parts: two 25 μL aliquots served as input controls for WB and RT-qPCR, while the rest was diluted and incubated with anti-Flag agarose beads at 4 ℃ overnight with rotation. Beads were washed three times with pre-cooled PBS and split into two fractions for WB analysis and TRIzol-based RNA isolation. Extracted RNAs were reverse-transcribed and subjected to RT-qPCR quantification. Relative enrichment of target RNAs was normalized to corresponding input samples.

### RNA pull-down assay

The pull-down assay was performed using the F2-RNA Pull-Down Kit (FITGENE, FI8721). Briefly, HEK293T cells expressing RIG-I protein were lysed in 1 mL RIPA lysis buffer. 100 µL lysate was used as input, and the remaining 900 µL was split equally into nine aliquots. Nine F2-tagged IVT full-length Vif RNA probes (Vif without m5C, Vif with m5C, Vif mut all, C5049A, C5056A, C5075A, C5082A, C5098A, C5100A) were generated via in vitro transcription. Each 3 µg RNA was denatured at 95°C for 3 min, chilled on ice for 1 min, mixed with RNA structure buffer and RNase inhibitor, and refolded at room temperature for 30 min. Each RNA probe was incubated with pre-washed magnetic beads on a rotator at room temperature for 30 min. Beads were captured by magnetic stand, washed twice with wash buffer, and incubated with separate cell lysate aliquots at 4 °C for 4 h with rotation. After repeated washing, bound proteins were eluted in 1 × SDS loading buffer at 95°C for 5 min, and eluates were analyzed by western blotting.

### Electrophoretic Mobility Shift Assay (EMSA)

5′ biotin-labeled RNA probes (Sangon Biotech) were incubated with recombinant RIG-I protein (MCE, HY-P72170) in the binding buffer from Beyotime Gel-Shift kit (GS005) at 23 °C for 30 min. Colorless EMSA loading buffer was mixed with the reaction system, and samples were loaded onto the pre-electrophoresed gel followed by electrophoresis at 100 V for 90 min. Separated RNA-protein complexes were transferred to a membrane (Yeasen, 60144ES76P) in 0.5 × TBE at 60 V for 60 min. The membrane was blocked with blocking buffer containing 5% BSA and 0.02% Tween 20 at room temperature for 30 min, then incubated with HRP-streptavidin conjugate for 45 min at room temperature. Target bands were visualized with ECL chemiluminescence reagent. RNA probe sequences are shown below:

None-m5C RNA: Biotin-AUGGAAAACAGAUGGCAGGUGAUGAUUGUGUGGCAAGUAGACAGGAUGAGGAUUAACACUGGA-3’

m5C RNA: Biotin-AUGGAAAA/i5MerC/AGAUGG/i5MerC/AGGUGAUGAUUGUGUGG/i5MerC/AAGUAGA/i5MerC/AGGAUGAGGAUUAA/i5MerC/A/i5MerC/AUGGA-3’

### RNA stability assay

HEK293T and NSUN2-knockout HEK293T cells were seeded into 12-well plates at a density of 0.5 million cells per well. Twenty-four hours later, cells were transfected with 1 μg pNL4–3 R ⁺ E^−^ luc plasmid per well. At 12 h post-transfection, culture medium was replaced with fresh medium supplemented with 1 μg/mL actinomycin D (Act D). Cells were harvested at 0, 3, 6, and 9 h after Act D treatment, and total RNA was isolated using TRIzol reagent. Extracted RNA was reverse-transcribed and analyzed via RT-qPCR to detect Gag and Tat mRNA abundance, with GAPDH as the internal control. Relative mRNA levels were normalized to the value of the 0 h group, which was defined as 1.0.

### Primary human CD4 ⁺ T cell isolation, stimulation and HIV-1 infection

Cryopreserved human PBMCs were thawed and cultured in RPMI 1640 medium with 10% FBS and 1% penicillin-streptomycin for 24 h prior to CD4 ⁺ T cell isolation. Six-well plates were pre-coated with 10 μg/mL anti-CD3 antibody diluted in PBS at 37 ℃ for 2 h. PBMCs were centrifuged and adjusted to 1 × 10⁸ cells/mL, then CD4 ⁺ T cells were purified via the MojoSort kit following manufacturer’s instructions. Isolated CD4 ⁺ T cells were seeded into coated plates and stimulated with 1 μg/mL anti-CD28 antibody plus 20 ng/mL IL-2. Three days later, cells were cultured at 0.5-1 × 10⁶ cells/mL with 20 ng/mL IL-2.

For HIV-1 infection, activated CD4 ⁺ T cells were seeded at 1 million cells per well, incubated with HIV-1 stock containing 500 pg virions, and centrifuged at 1,200 rpm for 2 h to boost viral attachment. After another 2 h incubation at 37 ℃, medium was refreshed with IL-2-supplemented RPMI 1640. Cell pellets and supernatants were collected at 24, 48, 72 and 96 h post-infection, with uninfected 0 h cells as blank controls.

### HIV-1 p24 ELISA assay

Cell supernatants were cleared by centrifugation at 1,000 × g for 20 min. HIV-1 p24 levels were measured with JL19101 sandwich ELISA kit (JONLNBio, JL19101) per manufacturer’s instructions. Briefly, reagents were equilibrated to room temperature, and standard serial dilutions (1000–15.6 pg/mL) were prepared. Samples and standards (100 μL/well) were incubated at 37 ℃ for 60 min, followed by biotinylated antibody incubation, repeated washing, streptavidin-HRP incubation, TMB color development and termination. Absorbance at 450 nm was detected immediately. Duplicate OD values were blank-corrected, and sample p24 concentrations were calculated via four-parameter logistic standard curve with dilution factors adjusted as required.

### Data analysis

All data are representative of three or more independent experiments and analyzed by two-tailed unpaired *t*-test or one-way ANOVA. Data are presented as the mean ± SEM. Statistical significance was defined as follows: NS, not significant (P > 0.05); *P < 0.05; **P < 0.01; ***P < 0.001; ****P < 0.0001.

## Supporting information

S1 Raw Image Full uncropped raw blot images corresponding to all main figures. This file contains all original, uncropped, and unadjusted Western blot and dot blot raw images corresponding to all main figures.
(PDF)
