# Supplementary material for: NSUN2-mediated m5C modification of HIV-1 RNA enables evasion of RIG‑I‑dependent innate immunity
Source: PLoS Pathog. 2026 Sep 25;22(9):e1014611. doi: 10.1371/journal.ppat.1014611 (PMC13614588; doi:10.1371/journal.ppat.1014611)

Figure 1

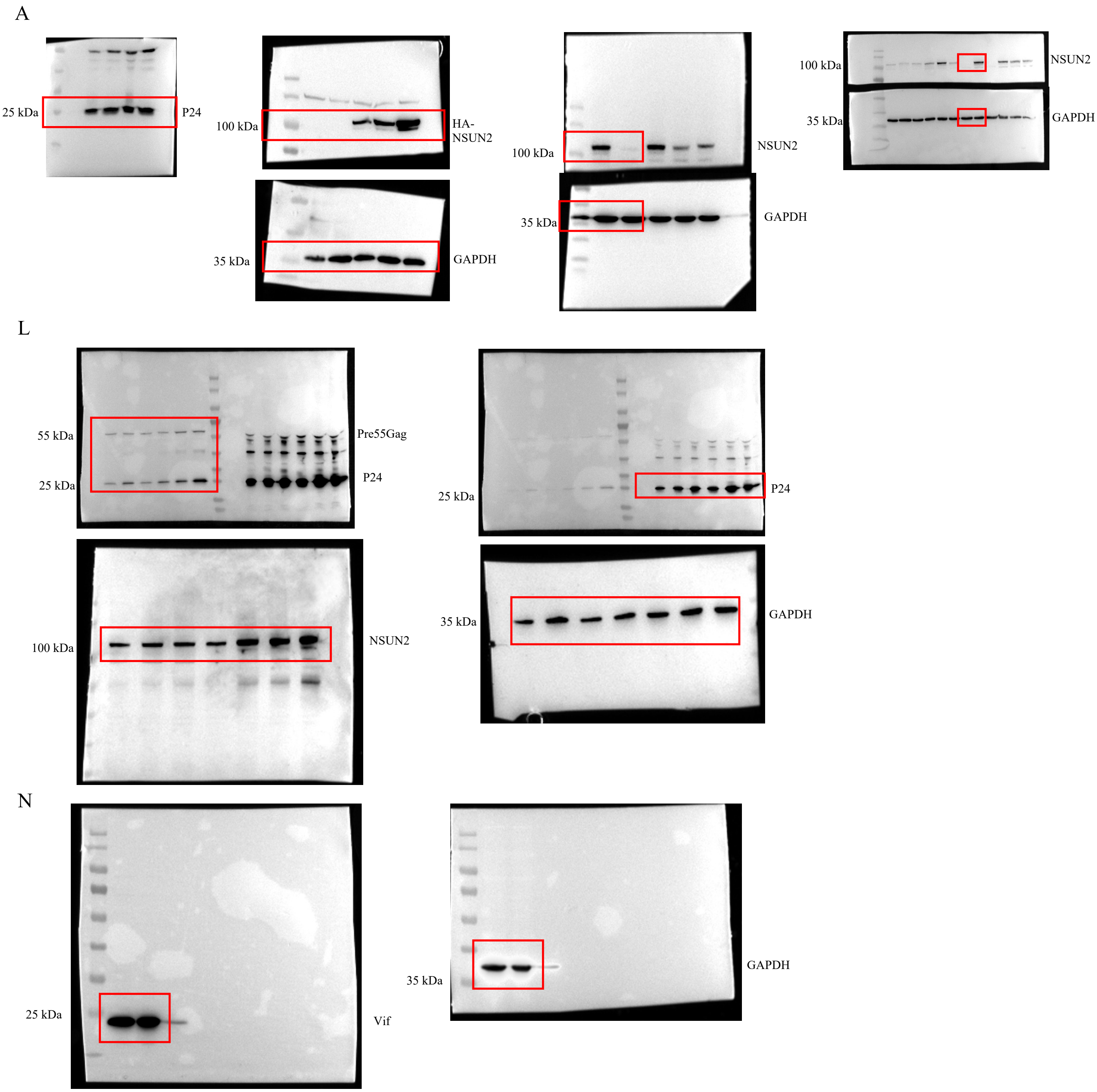

Figure 2

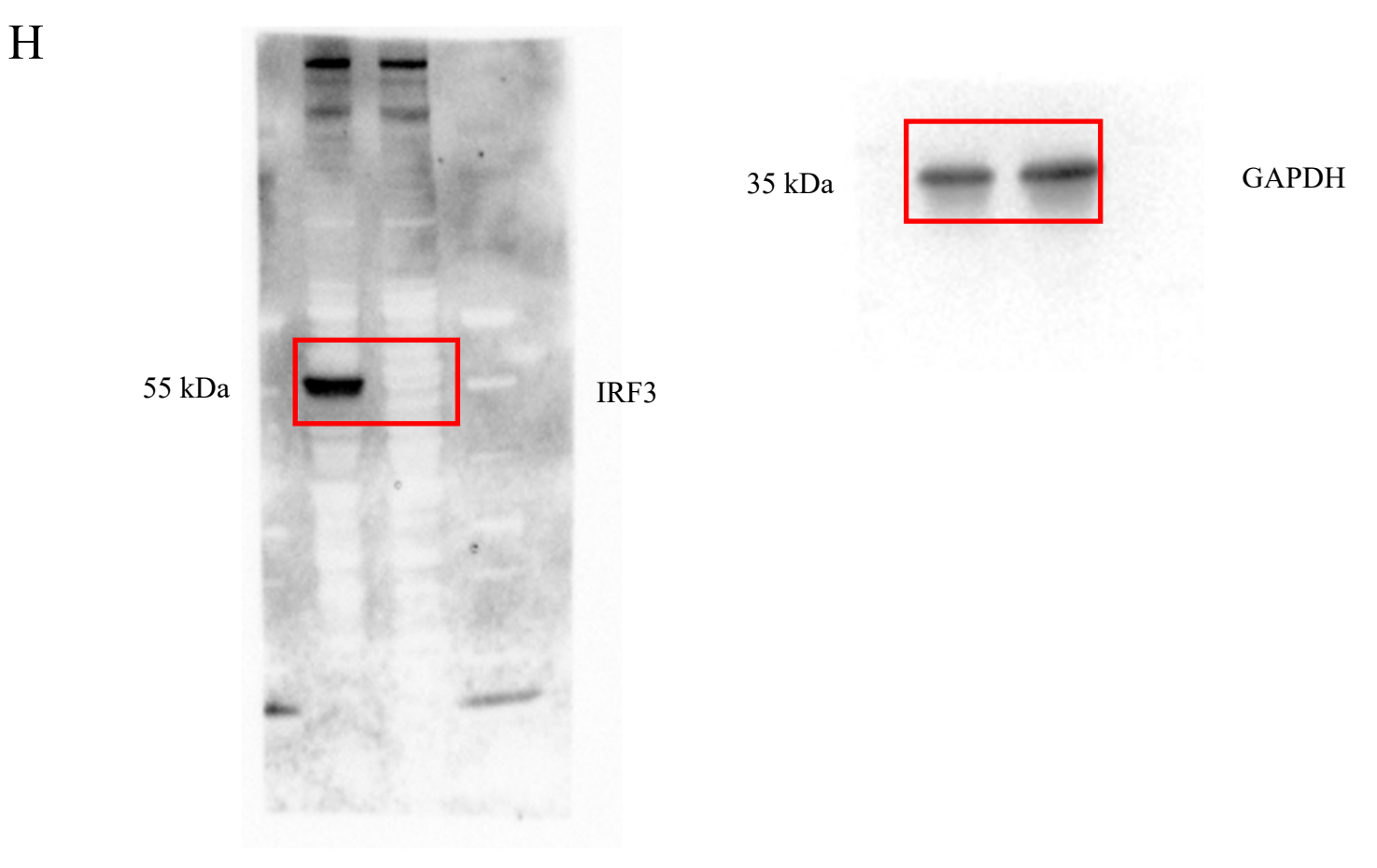

Figure 3

A

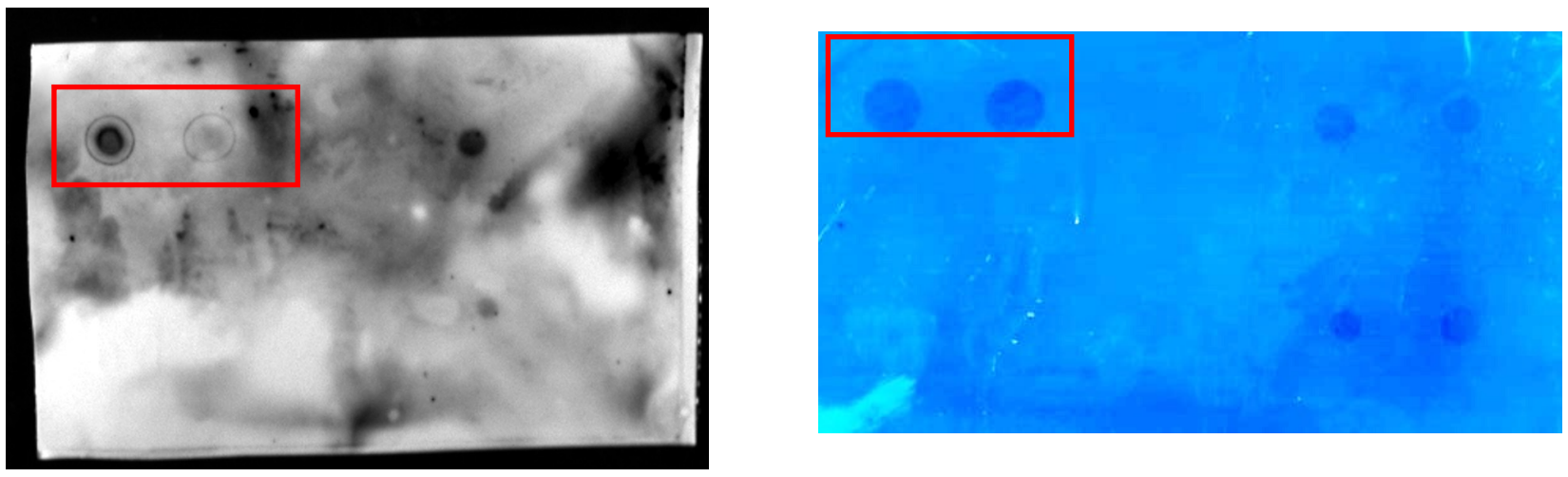

D

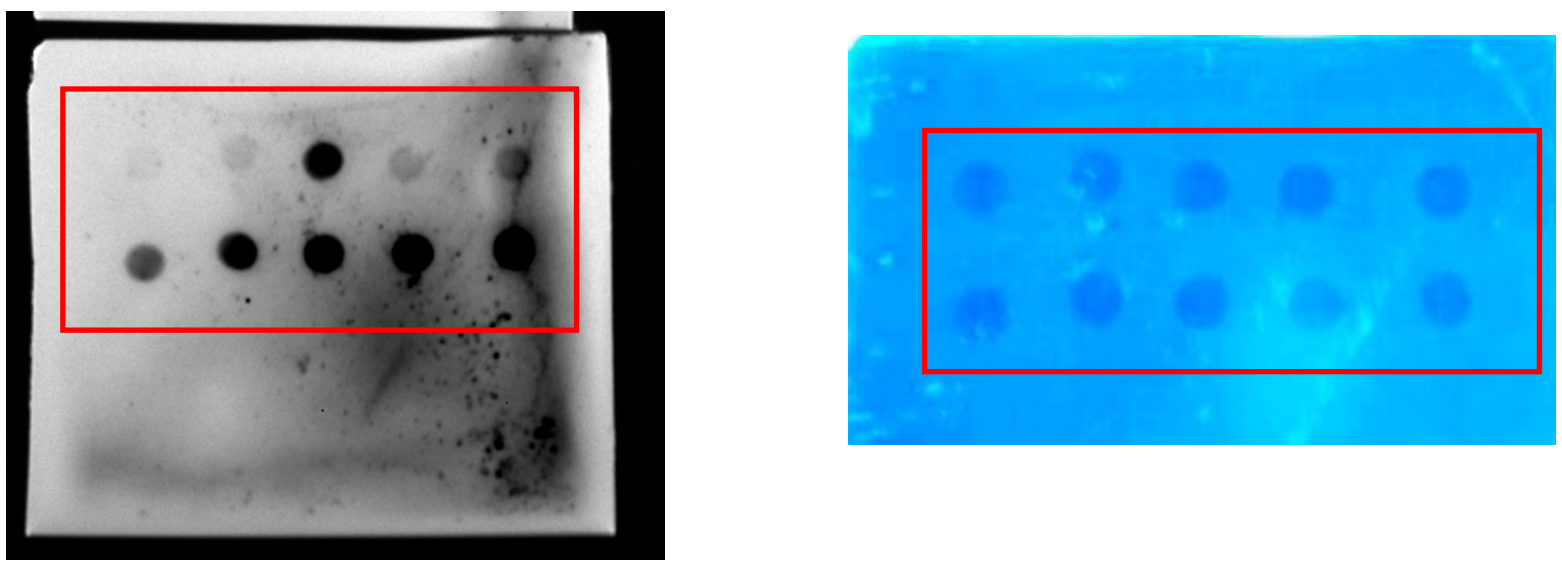

Figure 4

A

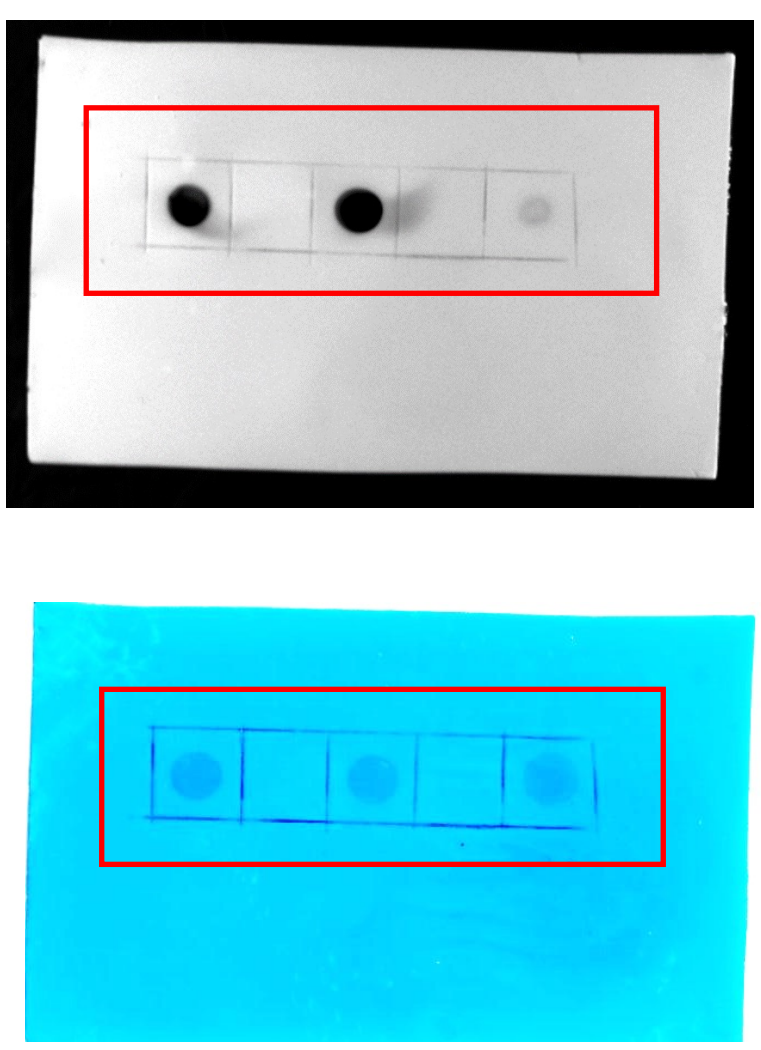

D

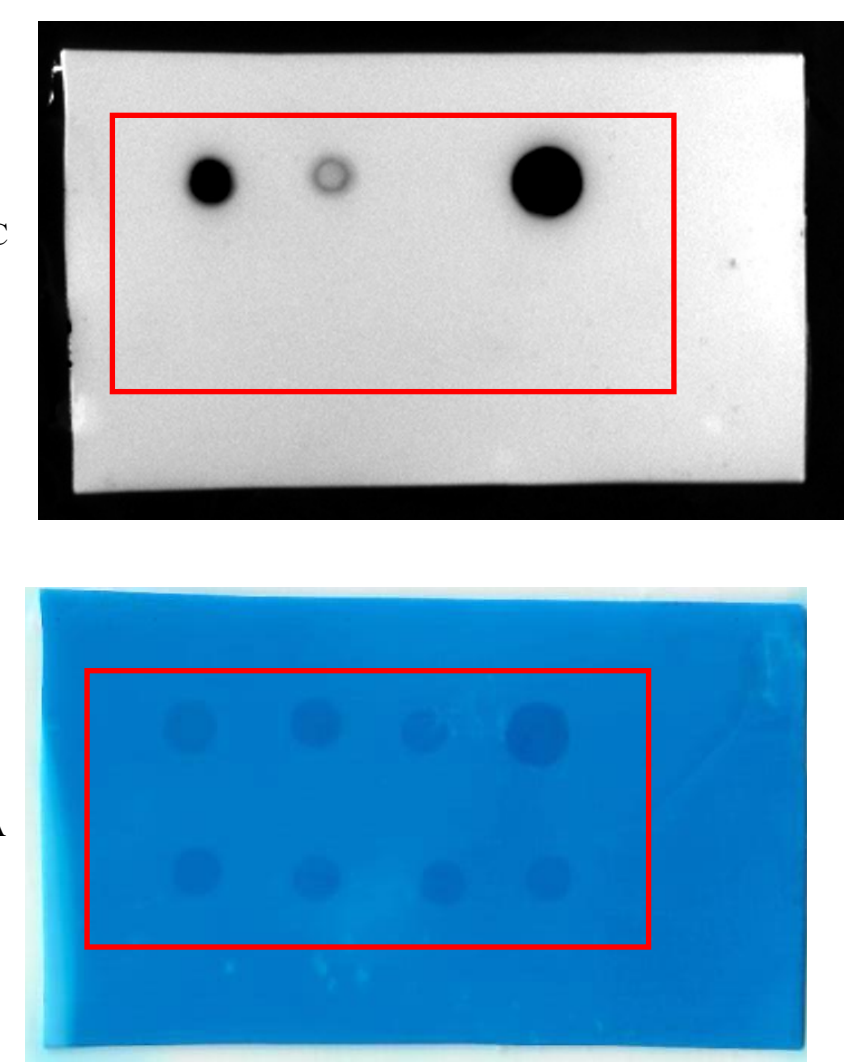

Figure 5

A

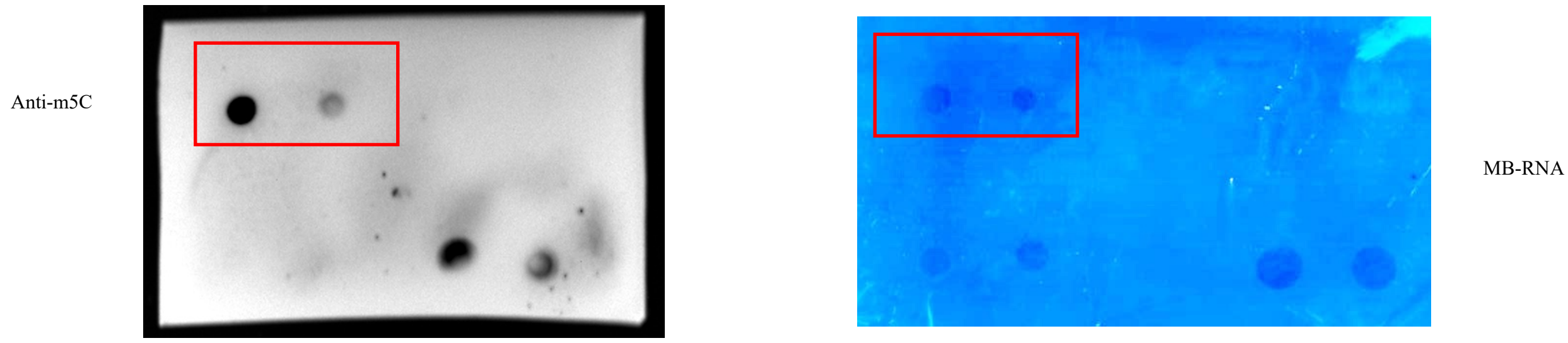

Figure 6

A

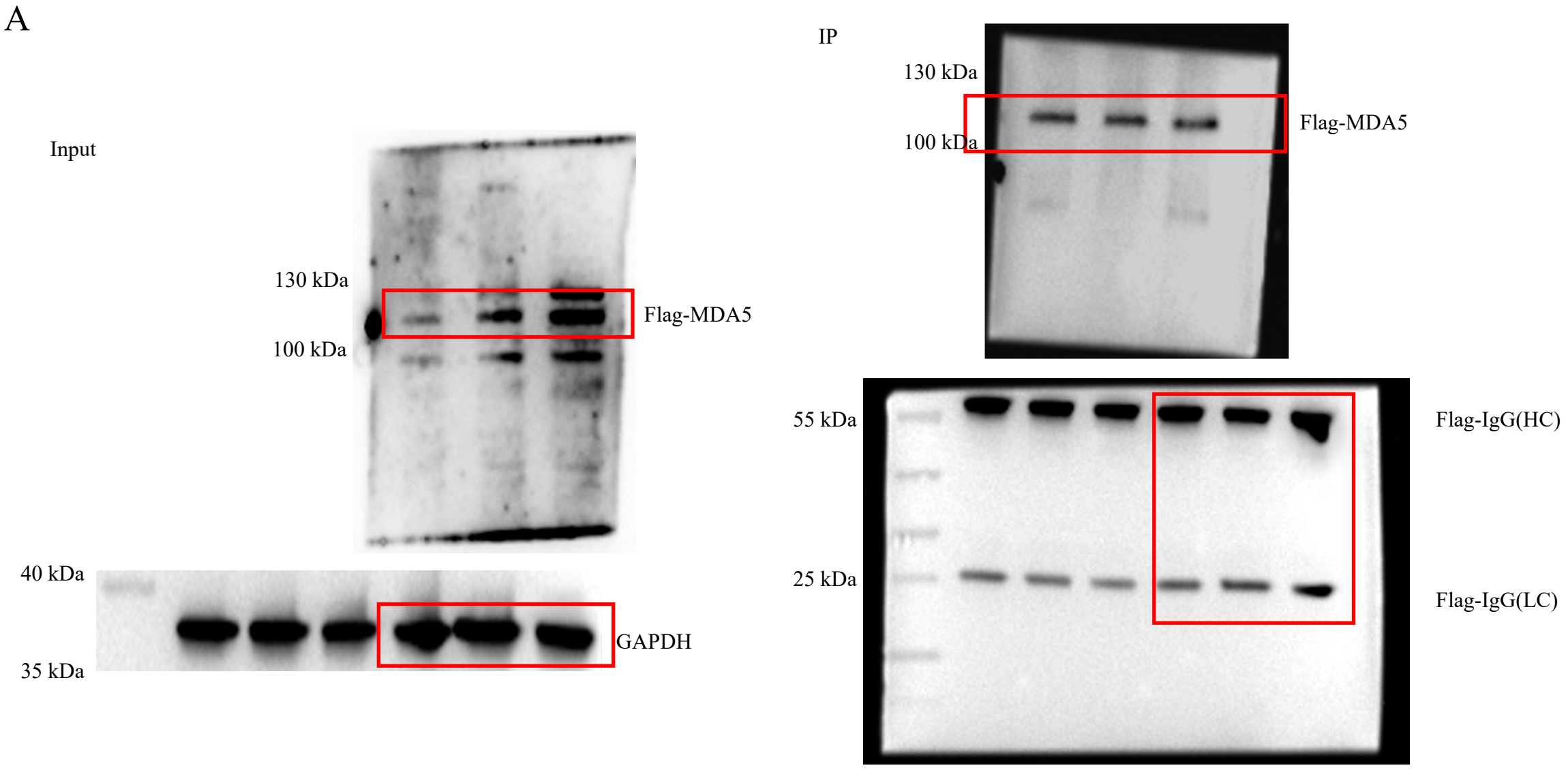

C

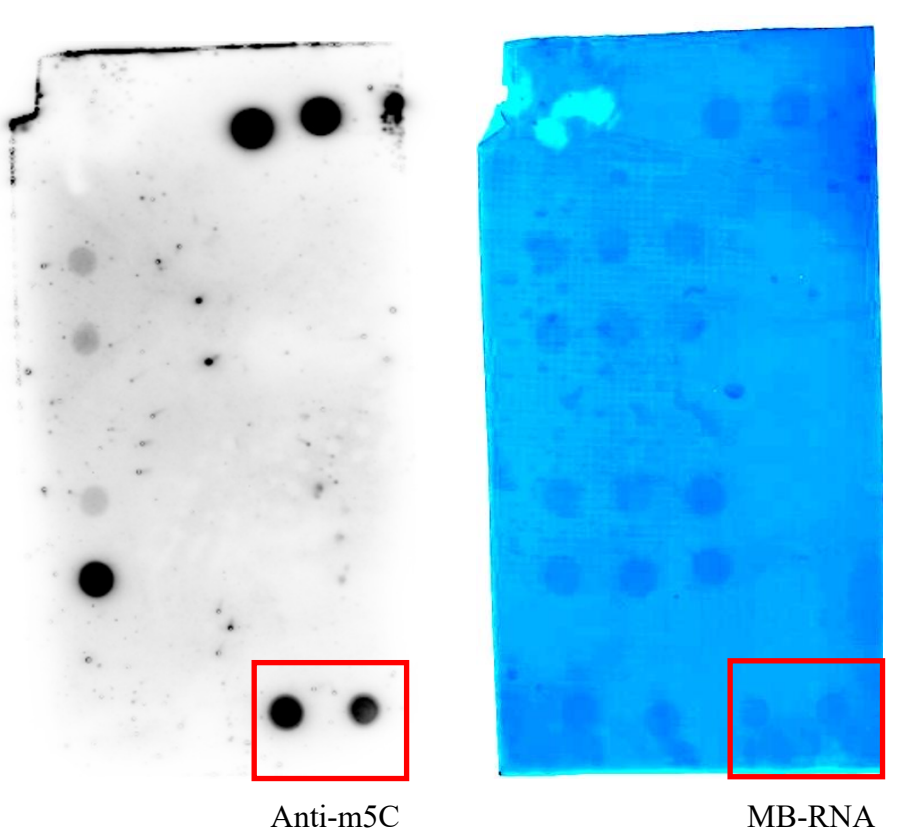

D

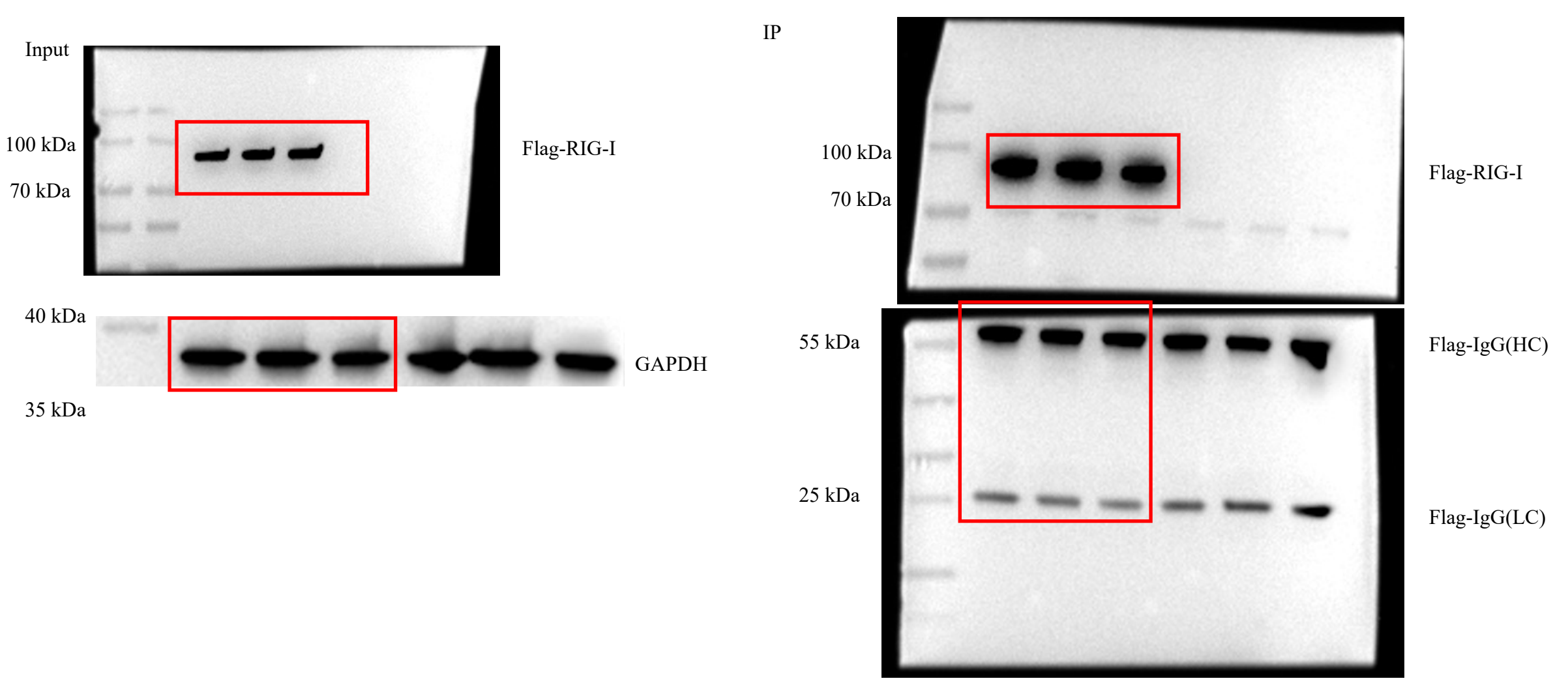

Figure 6

F

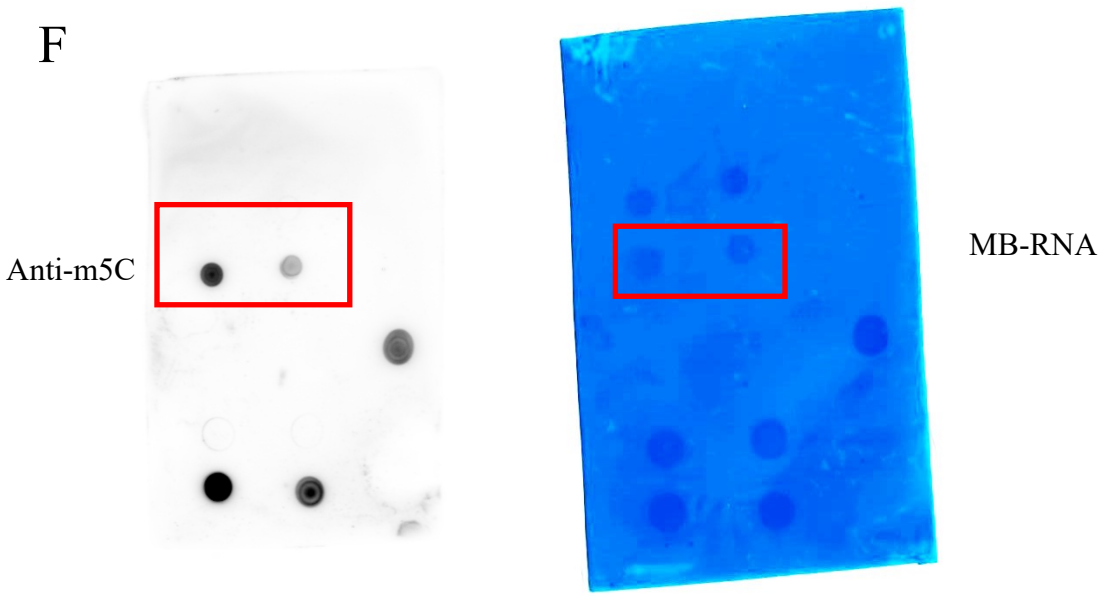
Anti-m5C
MB-RNA

H

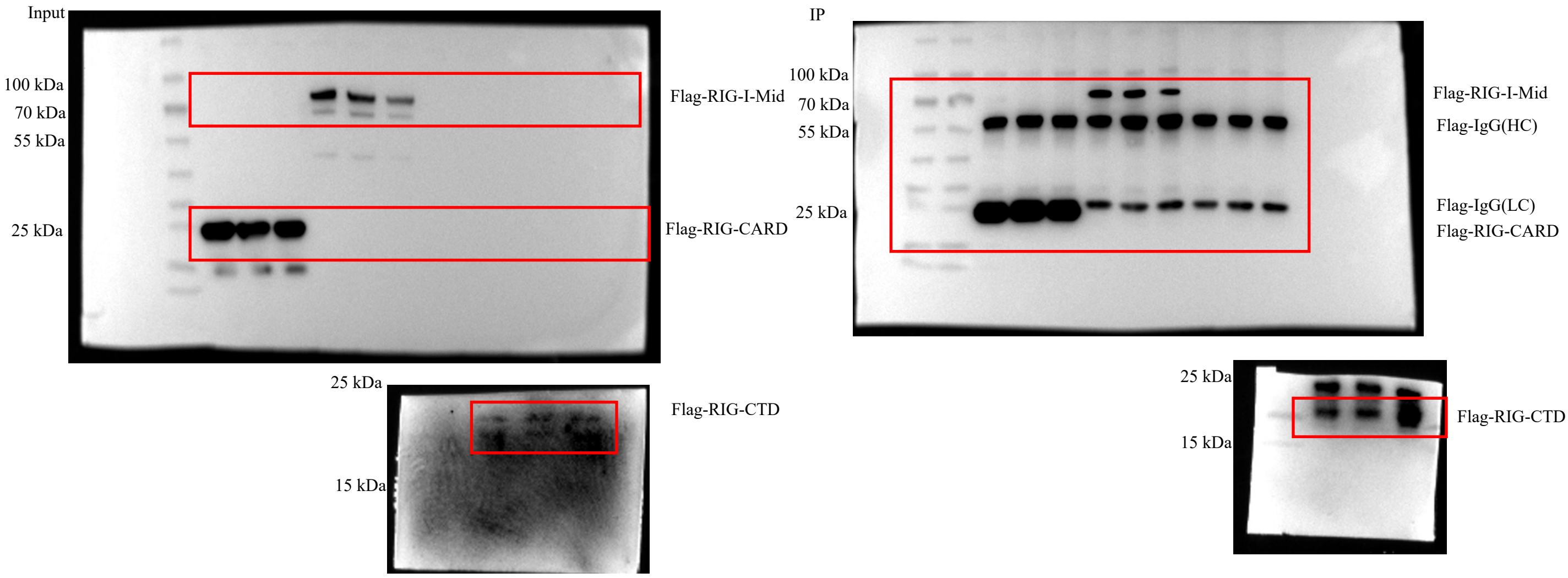
Input
100 kDa
70 kDa
55 kDa
25 kDa
Flag-RIG-I-Mid
Flag-RIG-CARD
25 kDa
15 kDa
Flag-RIG-CTD
IP
100 kDa
70 kDa
55 kDa
25 kDa
Flag-RIG-I-Mid
Flag-IgG(HC)
Flag-IgG(LC)
Flag-RIG-CARD
25 kDa
15 kDa
Flag-RIG-CTD

J

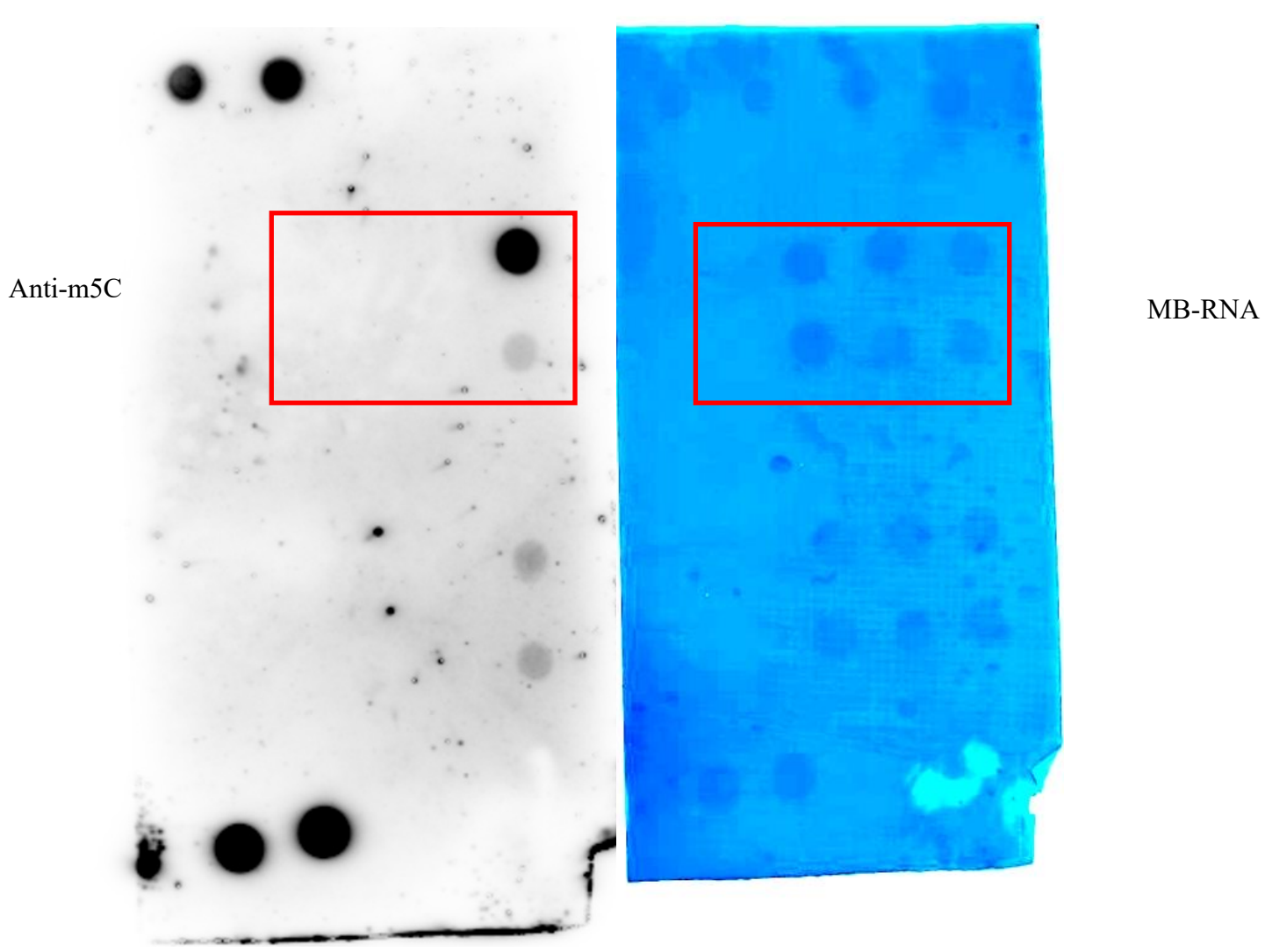
Anti-m5C
MB-RNA

Figure 6

K

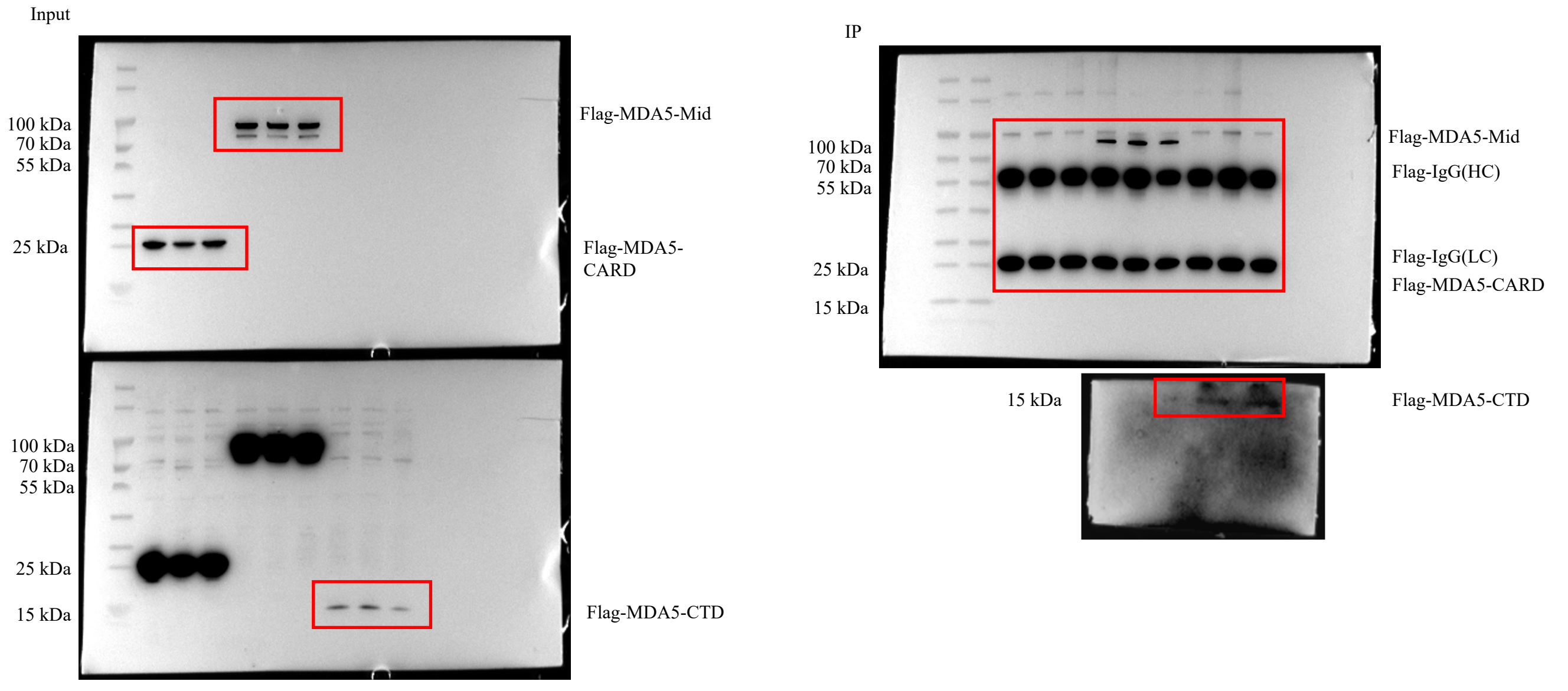

M

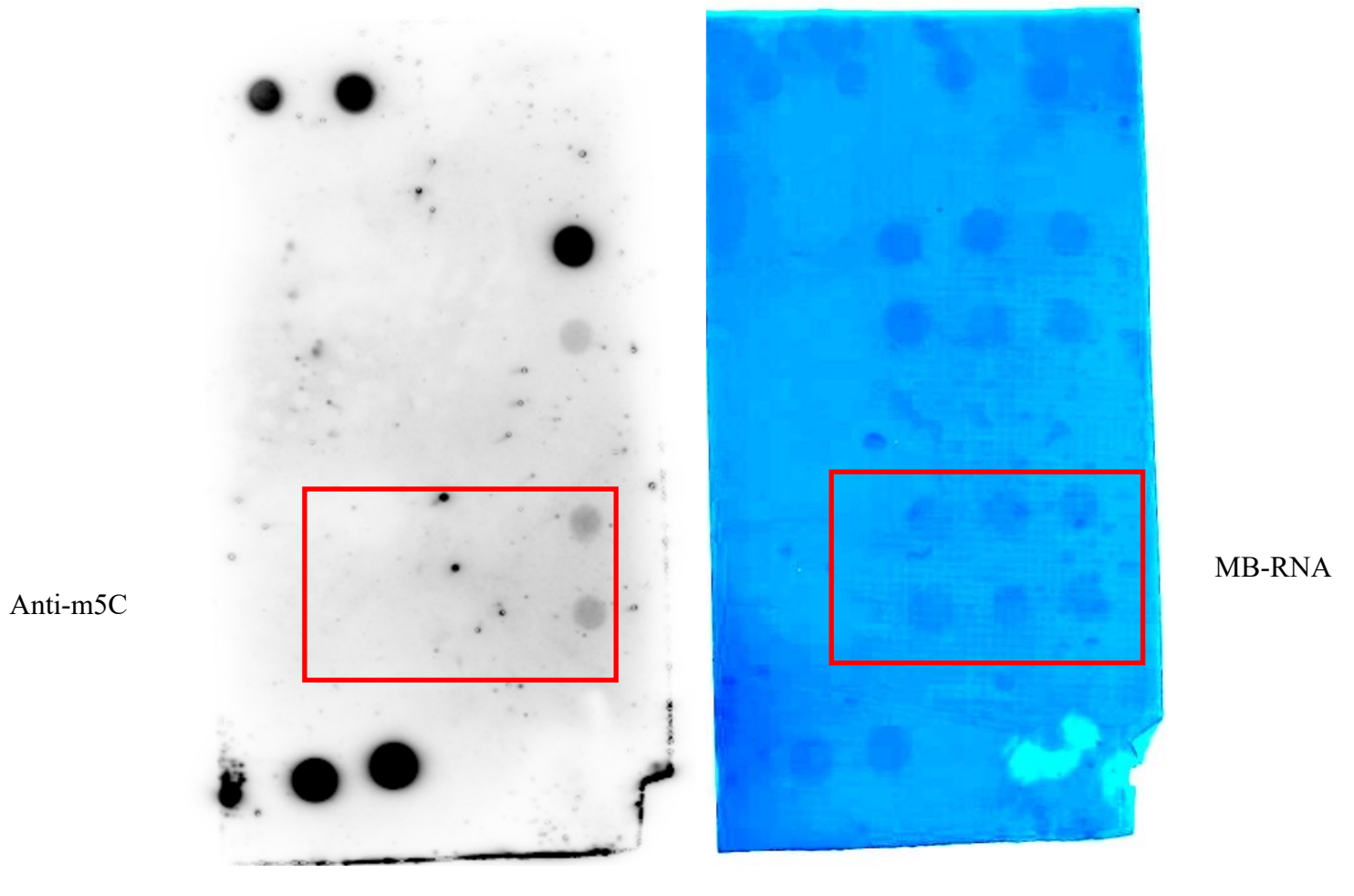

S

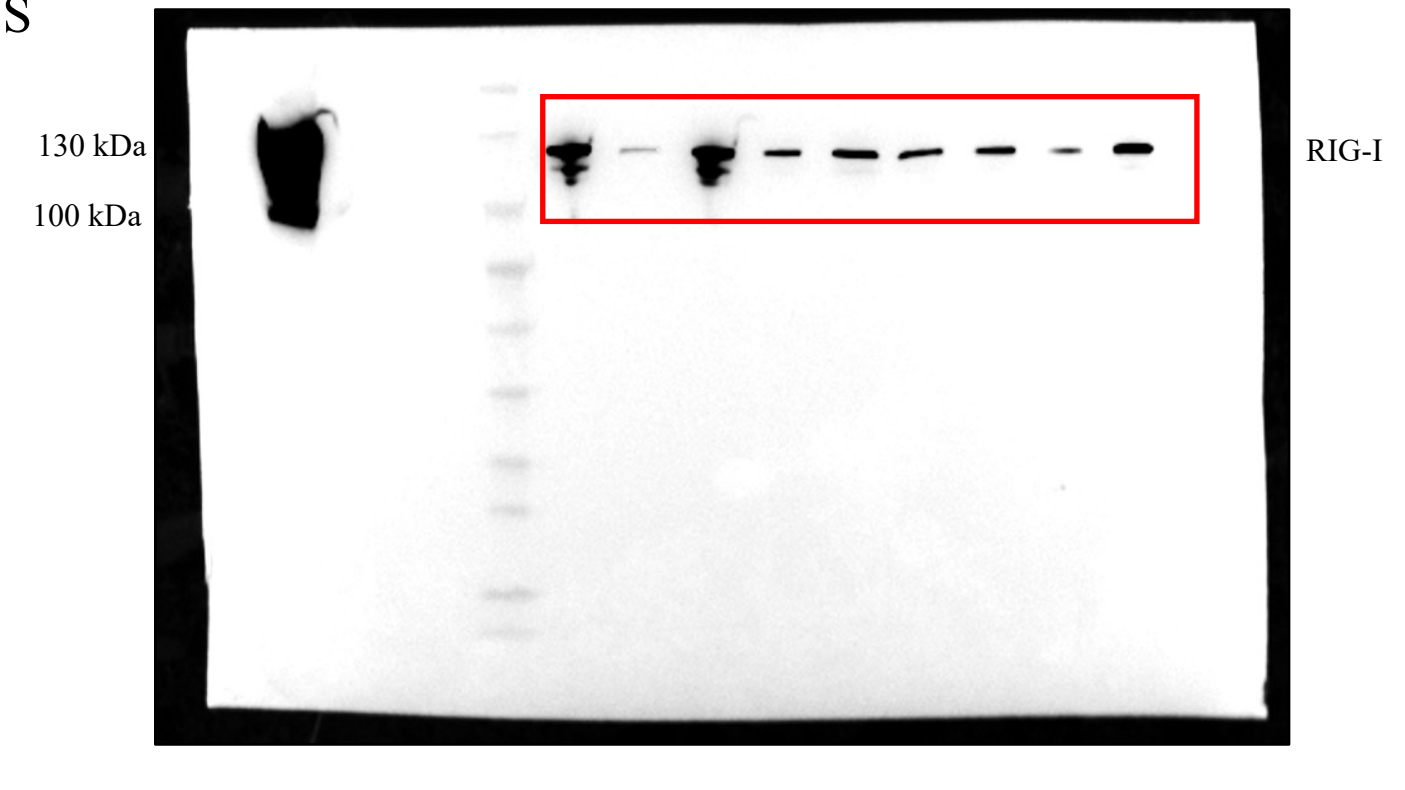

T

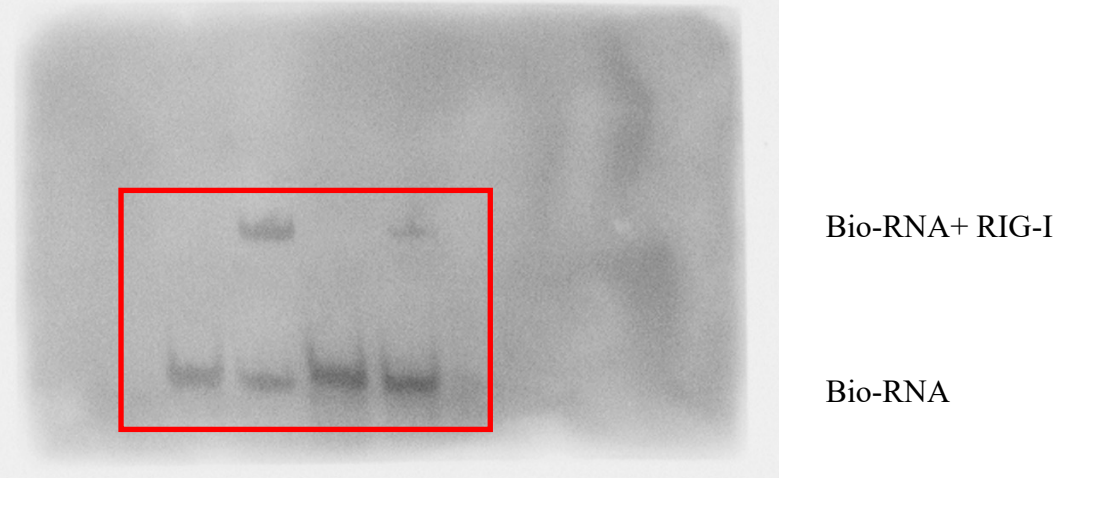

Supplement: Supplementary file 1 [file ppat.1014611.s001.pdf]
