# Supplementary material for: NSUN2-mediated m5C modification of HIV-1 RNA enables evasion of RIG‑I‑dependent innate immunity
Source: PLoS Pathog. 2026 Sep 25;22(9):e1014611. doi: 10.1371/journal.ppat.1014611 (PMC13614588; doi:10.1371/journal.ppat.1014611)
Supplement: Supplementary file 2 [file ppat.1014611.s003.docx]

**Reviewer comments:**

**Reviewer #1:**

In this manuscript, Xu et al. report a novel and important mechanism by which HIV-1 hijacks the host m5C methyltransferase NSUN2 to install m5C modifications on viral genomic RNA, thereby blunting RIG-I–dependent innate immune sensing. The study is well conceived and thoughtfully executed. The data are solid, internally consistent, and supported by multiple complementary approaches, and the authors’ conclusions are well justified by the experimental evidence. Overall, the work significantly advances our understanding of how viral RNA epitranscriptomic regulation contributes to innate immune evasion and highlights NSUN2-dependent m5C modification as a potentially exploitable host pathway in antiviral immunity.

The manuscript is clearly written, the experimental logic is easy to follow, and the findings should be of broad interest to readers in viral pathogenesis, RNA biology, and innate immunity. I therefore consider this work suitable for publication in PLOS Pathogens after minor revision.

Answer: We sincerely appreciate your positive evaluation and valuable comments on our work. We have addressed all the suggested points and revised the manuscript as detailed below. All page and line numbers mentioned below correspond to the marked-up version of the manuscript.

**Major Comments:**

1.Previous studies have shown that NSUN2-mediated m5C modification regulates the stability of host RNAs. In the current study, the focus is on RIG-I sensing and immune evasion. Please clarify whether NSUN2-dependent m5C modification also affects HIV-1 RNA stability (e.g., RNA half-life/decay assays), and discuss how any stability effects might contribute to the observed phenotypes.

Answer: Thank you for your valuable comment. We performed actinomycin D chase assays to measure the decay rate of HIV-1 RNA. Briefly, WT, NSUN2-KO, and NSUN2-overexpressing cells were transfected with HIV-1 pNL4-3 plasmid. Twenty-four hours post-transfection, actinomycin D was added to block de novo transcription, and viral mRNA was harvested at sequential time points for analysis. Our results demonstrate that NSUN2-dependent m5C modification has no detectable impact on HIV-1 RNA stability (newly added Figure 1O and 1P; pages 8, lines 146-154). As suggested, we have also included relevant discussion regarding the potential role of RNA stability in shaping the observed phenotypes (page 8, lines 154-158).

2.Most key experiments were performed in HEK293T, A549, and Jurkat cells. However, monocytes and macrophages are major physiological targets of HIV-1 and are highly relevant to innate immune sensing. Please validate the key findings in primary monocytes or monocyte-derived macrophages to confirm that NSUN2-mediated m5C modification promotes HIV-1 immune evasion in physiologically relevant cells.

Answer: We appreciate this important comment. We have validated our key phenotypes in two physiologically relevant cell models, including THP-1-derived macrophages and primary human CD4⁺ T cells.

For the first assay, THP-1 monocytes were differentiated into macrophages via PMA stimulation. These differentiated macrophages were transfected with genomic RNA purified from HIV-1 virions secreted by WT or NSUN2-KO producer cells. Cellular RNA was collected 24 h post-transfection to measure innate immune activation, with poly (I:C) as a positive control. Consistently, viral RNA extracted from NSUN2-KO-derived virions triggered markedly stronger innate immune responses (newly added Figure 4E-G; pages 12-13, lines 250-258).

In the second experiment, primary CD4⁺ T cells were infected with authentic HIV-1 generated by WT or NSUN2-KO producer cells. Cellular RNA was harvested for qPCR quantification of *IFNA1*, *IFNB1* and viral *Gag* mRNA. Meanwhile, cell culture supernatants were collected to measure viral loads. Our results reveal that virions lacking NSUN2-dependent m5C modifications trigger robustly enhanced innate immune responses and display greatly impaired replication. All these supplementary data have been integrated into the revised manuscript (newly added Figure 5F-I; pages 14-15, lines 292-305).

**Minor Comments：**

1.Statistical asterisks in the figures vary in size; please standardize them for readability.

Answer: We appreciate this careful comment. We have standardized the size of all statistical asterisks across all figures in the revised manuscript for better readability.

2.In the Figure 2 legend, please correct NSUN KO to NSUN2 KO (line 356).

Answer: We appreciate this reminder. We have corrected NSUN KO to NSUN2 KO in the Figure 2 legend (line 509) as requested.

3.In the Materials and Methods, please correct ware to were (line 537).

Answer: We appreciate this comment. We have fixed the typo ware to was (line 731) as suggested.

**Reviewer #2:**

This manuscript investigates how HIV-1 uses a chemical modification called m5C on its RNA to hide from the host immune system. The authors show that a host enzyme, NSUN2, adds m5C marks to HIV-1 genomic RNA. These marks prevent the immune sensor RIG-I from detecting the viral RNA, which in turn reduces the production of type I interferons (IFN-I). When NSUN2 is knocked out in virus-producing cells, the resulting viruses carry less m5C on their RNA, trigger a stronger immune response, and replicate less efficiently. The authors further pinpoint RIG-I (not MDA5), and specifically its C-terminal domain (CTD), as the sensor that distinguishes between m5C-modified and unmodified viral RNA.

Overall, the topic is timely and the central findings are of interest. However, several experimental and conceptual concerns need to be addressed before this work can be considered for publication.

Answer: We greatly appreciate your thorough summary and objective evaluation of our manuscript. We fully recognize the experimental and conceptual concerns you raised, and we have systematically supplemented and revised corresponding experiments and discussion sections point by point in accordance with all your suggestions to strengthen our conclusions. All page and line numbers mentioned below correspond to the marked-up version of the manuscript.

**Major Comments：**

1. NSUN2 knockout affects many things beyond m5C on HIV-1 RNA. How can we be sure the effects are specific?

The study uses NSUN2 knockout as the main tool to study m5C’s role in immune evasion. The problem is that NSUN2 does not only modify HIV-1 RNA — it also adds m5C to many cellular RNAs, including tRNAs and mRNAs. Knocking out NSUN2 will therefore cause widespread changes in the cell (e.g., altered tRNA stability, translation, and gene expression), and some of these changes could indirectly affect viral replication or immune signaling.

The authors do include a good control in Figure 3D–E: they rescue NSUN2 KO cells with wild-type NSUN2 or a catalytically dead mutant and show that only the active enzyme restores normal IFN levels. However, this rescue is only done for cellular RNA. The same rescue experiment should be repeated in the HIV-1 infection context (i.e., in the experiments shown in Figures 4–6) to confirm that the immune evasion phenotype is truly due to NSUN2’s catalytic activity on viral RNA, rather than other indirect consequences of NSUN2 loss.

Answer: We appreciate this insightful and critical comment, which we fully agree is an important consideration for interpreting our results.

To exclude confounding effects arising from loss of m5C modification on cellular RNAs, we isolated viral genomic RNA purified from virions produced in NSUN2-KO producer cells and transfected this m5C-deficient viral RNA into WT cells. We observed robustly enhanced innate immune activation upon transfection, which directly demonstrates that the lack of NSUN2-dependent m5C marks on viral RNA drives stronger immune signaling independent of cellular RNA m5C status (Figure 4A-C, 5J-L, newly added Fig. 4E-G).

We acknowledge that NSUN2 knockout may indirectly modulate viral replication by altering m5C levels of host cellular RNAs. We have expanded the Discussion section to address this potential limitation and indirect cellular effects (pages 21-22, lines 446–457).

As requested, we have performed the rescue experiment in the context of HIV-1 infection. Briefly, NSUN2-KO producer cells were transfected with WT NSUN2 or its catalytically inactive C321A/I302A mutant prior to generating VSV-G pseudotyped HIV-1 virions. We then infected target cells with these viruses and measured innate immune responses. Viral RNA m5C levels were validated via dot blot analysis. Consistent with our earlier observations, these results confirm that the immune evasion phenotype relies specifically on the catalytic activity of NSUN2 toward viral RNA (newly added Figure 4D; page 12, lines 240–249).

2. All experiments use immortalized cell lines

The study relies entirely on transformed cell lines (HEK293T, HeLa, A549, and Jurkat). While these are convenient and widely used, they do not fully represent the cells that HIV-1 naturally infects in the human body, such as primary CD4+ T cells and macrophages. These primary cells have different levels of RIG-I and MDA5, and their innate immune signaling can behave quite differently. To make the findings more physiologically relevant, the authors should validate at least the key results (e.g., stronger IFN induction by m5C-deficient virus, reduced viral replication) in primary CD4+ T cells or monocyte-derived macrophages.

Answer: We greatly appreciate this valuable comment highlighting the physiological relevance of our cellular models. As suggested, we have validated our core phenotypes in two physiologically relevant cell systems: PMA-differentiated THP-1 macrophages and primary human CD4⁺ T cells.

For the first assay, THP-1 monocytes were differentiated into macrophages with PMA treatment. These differentiated macrophages were transfected with genomic RNA purified from HIV-1 virions produced by WT or NSUN2-KO producer cells. Cellular RNA was harvested 24 h post-transfection to quantify innate immune activation, with poly (I:C) included as a positive control. Consistent with our data from immortalized cell lines, viral RNA isolated from NSUN2-KO-derived virions induced markedly stronger innate immune responses (newly added Figure 4E-G; pages 12-13, lines 250-258).

In the second experiment, primary human CD4⁺ T cells were infected with authentic HIV-1 virions generated by WT or NSUN2-KO producer cells. We extracted cellular RNA for qPCR detection of *IFNA1*, *IFNB1* and viral Gag transcripts, and collected cell culture supernatants to quantify viral loads by p24 ELISA. Consistent with our earlier observations in transformed cell lines, loss of NSUN2-mediated m5C modifications on viral RNA triggered robustly elevated innate immune signaling and greatly attenuated viral replication in primary CD4⁺ T cells. All these supplementary data have been incorporated into the revised manuscript (newly added Figure 5F-I; pages 14-15, lines 292-305).

3. The Vero vs. A549 comparison (Fig. 2H) does not cleanly demonstrate IFN-I dependence.

The authors compare viral infectivity in Vero cells (which cannot produce IFN) and A549 cells (which can) and conclude that the benefit of m5C modification depends on IFN-I signaling. The issue is that Vero and A549 cells are completely different cell types, so many factors beyond IFN competence could explain the observed differences.

A cleaner experiment would be to use the same cell type under two conditions: one where IFN signaling is intact, and one where it is blocked. This could be achieved by treating cells with an anti-IFNAR blocking antibody, a JAK inhibitor (e.g., ruxolitinib), or by using IFNAR-knockout cells alongside their wild-type counterparts.

Answer: We appreciate this important comment and fully recognize the limitation of comparing two distinct cell lines (Vero and A549) to verify IFN-I dependence.

To resolve this issue, we removed the data from Vero and A549, and performed parallel experiments in HeLa cells and HeLa IRF3-knockout cells. Consistent with our previous conclusions, the loss of IFN signaling in IRF3-deficient cells eliminated the difference in viral infectivity caused by viral m5C modification, supporting our conclusion that NSUN2 mediated m5C modifications promote viral replication through intact type I interferon responses. (revised Figure 2H-I; page 10, lines 192-202).

**Minor Comment****s：**

1. Typo in Figure 1 legend.

Line 346: “NSUN2-overexpressin” should be corrected to “NSUN2-overexpressing.”

Answer: We appreciate this comment. We have fixed the typo “NSUN2-overexpressin” to “NSUN2-overexpressing” (line 489).

2. Labeling error in Figure 7.

The schematic model labels the knockout condition as “NSU2N KO” — this should be “NSUN2 KO.”

Answer: We appreciate your careful check. We have corrected the labeling typo “NSU2N KO” to “NSUN2 KO” in the schematic diagram of Figure 7.

3. The use of N1-methylpseudouridine in IVT RNA may confound interpretation.

In the in vitro transcription experiments, the authors replace UTP with N1-methylpseudouridine (N1-Me-Pseudo UTP; line 514). This modified nucleotide is itself well known to reduce innate immune activation (as noted in Ref. 41 of the manuscript). This raises a concern: if both the U and C positions are modified in the m5C-containing RNA, how can we confidently attribute the reduced IFN induction specifically to m5C? The authors should explain why they chose to use N1-methylpseudouridine and discuss whether this could complicate the interpretation of their m5C-specific results. Ideally, a parallel set of IVT RNAs using standard UTP (with and without m5CTP) should be included as a control.

Answer: We appreciate this careful and valuable comment, we agree that the use of N1-methylpseudouridine could complicate the interpretation of the IVT RNA experiments. We initially used N1-methylpseudouridine for in vitro transcription strictly following the manufacturer’s standard kit protocol. Nevertheless, this co-existing modification does not alter our core conclusion that m5C suppresses innate immune sensing, because N1-methylpseudouridine was incorporated equally into all IVT RNA samples, with m5C modification serving as the sole experimental variable between groups. As requested, we have generated parallel IVT RNA substrates with standard UTP (with or without m5CTP). These IVT RNA substrates reproduced our experimental phenotypes, further verifying that m5C modification independently suppresses type I interferon signaling. The data are shown below.

**Reviewer #3:**

In this study, Yuebo Xu et al investigates the role of the NSUN2-mediated m5C Modification of HIV-1 RNA in modulating the innate immune response during viral infection. Building on previous findings that progeny virus is upregulated in response to NSUN2, the authors identify RIG-I, the sensor for non-m5C RNA, as preferentially binding non-m5C viral RNA. Through a combination of biochemical and virological assays, the study proposes a novel mechanism by which a HIV-1 utilizes m5C modification of viral genomic RNA to evade host-mediated innate immune regulation. They show that NSUN2-mediated m5C modification is sufficient to modestly evade innate immune activation and enhance viral replication both in vitro and in vivo, with approximately a 5-fold increase in viral load. While the premise is compelling, the rigor and depth of experimental validation should be improved to fully support the authors' conclusions.

Answer: We sincerely appreciate your comprehensive evaluation and valuable feedback on our manuscript. We have carefully addressed all the raised concerns point by point, with detailed revisions and supplementary experimental data provided in the following responses. All page and line numbers mentioned below correspond to the marked-up version of the manuscript.

**Major Comments:**

1.While the manuscript presents interesting and valuable findings, the overall clarity and flow would benefit from revision. I recommend improving the transitions between experiments to best guide the reader through the narrative. Clarifying the rationale behind each experimental step and providing more author-driven insights into data interpretation would enhance the coherence and readability of the manuscript.

For example, in lines 75-79, the authors introduce the concept of how NSUN2 mediates m5C modification and m5C sites without providing sufficient context regarding their prevalence or immunomodulatory significance, both within and beyond the specific context of NSUN2. Including this background would help readers better understand the relevance and implications of these elements as they pertain to this study.

Answer: We thank the reviewer for this thoughtful and constructive suggestion. We agree that the clarity and flow of the manuscript would be improved by more explicit transitions between experimental sections and clearer explanation of the rationale behind each experimental step. In the revised manuscript, we have therefore strengthened the narrative structure throughout the Results sections. (lines 120-123, lines 132-133, lines 167-169, lines 200-202, lines 213-217, lines 240-242, lines 260-262, lines 273-276, lines 281-283, lines 306-309, lines 316-318).

As requested, we supplemented sufficient background information about the prevalence and immunomodulatory functions of m5C modifications to help readers grasp the broader context and significance of our work (pages 4-5, lines75-90).

2. Study Rigor - Descriptions of the study methodology should be expanded. It is unclear how HIV-1 infectivity(RLU) was processed to arrive at differential expression. How is the RIP experiment completed? What are the methods of the RIP experiment? The authors should provide details on how the data were calculated and normalized. Lastly, neither the figures nor the figure legends indicate sample sizes or whether the presented experiments have been reproduced independently,for example fig 1F,3A,6A.

Answer: We appreciate this valuable comment regarding the rigor of our experimental methods. We have supplemented comprehensive methodological details as requested. We expanded descriptions of HIV-1 infectivity data processing, fully elaborated the complete procedures for RIP assays, and specified the relevant data calculation and normalization strategies in the revised Methods section (page 30, lines 628-633; page 32, lines 679-681; page 34, lines 723-726; page 35, lines 745-746). Meanwhile, we have noted independent experimental replicates in figure legends (including figs. 1F, 3A, 6A) and Materials and methods section (pages 32-33, lines 681-682; page 33, line 692).

3. In Figures 1H and 1I, the authors conclude that NSUN2 overexpression enhances viral replication. However, this conclusion is primarily based on the detection of HIV-1 infectivity (RLU) levels as an indicator of viral infection. Given the role of NSNUN2 on the regulation of progeny viruses, to substantiate the claim that NSNUN2 genuinely increases viral replication, it would be important to assess additional viral RNA or the production of infectious viral proteins to provide a direct and quantitative assessment of viral replication and infectivity. Additionally, as shown by the blue line, which represents RLU changes following NSUN2 KO, no significant difference were observed in Figures 1H and 1I, Is this consistent with the findings?

Answer: We appreciate this constructive comment. The non-significant phenotypes observed in original Figures 1H and 1I are likely caused by two factors: endogenous NSUN2 in WT cells is sufficient to support viral RNA methylation during replication, and HEK293T cells secrete minimal IFN-β upon HIV-1 infection. Together, these features mask phenotypic changes triggered by NSUN2 overexpression. To address this limitation, we repeated the experiments using HeLa target cells with more robust innate immune signaling and have replaced the original figures with these new results in the revised manuscript (revised Figure 1H-J; page 7, lines 130-137).

As suggested, we quantified viral RNA and viral particle levels released from HeLa and NSUN2-overexpressing target cells. Both target cell lines were pre-transfected with VSV-G plasmid prior to pseudotyped HIV-1 infection. The data show that NSUN2 overexpression enhances viral production. Combined with our RLU infectivity data, these results collectively verify that NSUN2 overexpression enhances viral replication. All new datasets and descriptions are integrated into the revised manuscript (newly added Figure 1L-M; page 8, lines 137-141).

4.In Figure 2D, the values for the NSUN2 OE group are higher than those of the WT group, with poor reproducibility across three independent replicates. Furthermore, these results are inconsistent with the immune factor data presented in Figures 2A, 2B, and 2C. These data should be removed, or the experiment be repeated to further investigate this discrepancy.

Answer: We appreciate your careful review and valuable advice. Figures 2A-2C and 2D adopt different experimental systems. Figure 2D uses a GFP reporter system for IFN-β detection, with flow cytometry showing approximately 5% background signal in uninfected samples. Neither the WT nor NSUN2 overexpression groups activated the reporter, and all measured values in these two groups merely reflect background noise.

To avoid misleading readers, we have deleted NSUN2 overexpression data from Figures 2D, 2E and 2F (revised Figure 2D-F).

5. In Figure 6, the RIP assay confirms the interaction between RIG-I and viral RNA. If this result is not supported by additional experiments, such as RNA pull-down or RNA FISH, this result raises some concerns for me.

Answer: We appreciate this insightful comment. As suggested, we have supplemented both RNA pull-down and EMSA experiments to further validate the interaction between RIG-I and viral RNA. The new data demonstrate that RNA lacking m5C modification efficiently binds RIG-I, and we have provided detailed descriptions of these results in the revised manuscript (newly added Figure 6S-T; pages 17-18, lines 360-379).

**Minor Comments:**

1.In Figure 1F and 2E, the images should add scale bars.

Answer: We appreciate this comment. Corresponding scale bars have been added to the images in Figure 1F and Figure 2E in the revised figures.

2. In Figure 6C and 6F, lanes should be labeled with sample names.

Answer: We appreciate this comment. We have labeled all lanes with corresponding sample names in Figure 6C and 6F.

3. Line 132 describes “in a does-dependent manner”,but Figure 2B do not show a clear dose-response relationship. Should this describe be revised or omitted?

Answer: We appreciate this careful check. We have revised the statement for Figure 2B and removed the wording regarding in a dose-dependent manner (page 9, line 180).

**Reviewer #4:**

This study reveals a novel immune evasion mechanism whereby HIV‑1 recruits the host methyltransferase NSUN2 to install m5C modifications on its genomic RNA, thereby shielding the viral RNA from RIG‑I recognition and suppressing the type I interferon response. These findings hold considerable significance for both fundamental research and translational applications. Nevertheless, the evidence supporting the core mechanistic model requires further strengthening, particularly with respect to the identification of functional m5C sites and the direct molecular basis of RIG‑I discrimination. The following key experiments are recommended to consolidate the conclusions.

Answer: We appreciate your thorough evaluation and valuable suggestions on our core mechanism. We address each comment point-by-point below and have supplemented achievable experimental data and manuscript revisions to strengthen our conclusions. All page and line numbers mentioned below correspond to the marked-up version of the manuscript.

**Major Comments:**

Although the authors demonstrate global changes in m5C levels using NSUN2 knockout/overexpression and dot blot assays, direct evidence for specific m5C modification sites on HIV‑1 RNA (e.g., via bisulfite sequencing or m5C‑RIP‑seq) is lacking. The study merely cites previous work suggesting an m5C site in the overlapping Pol‑vif region, but this site has not been validated by sequencing in the HIV‑1 NL4‑3 background used herein. Moreover, the authors do not generate a site‑specific cytosine‑to‑adenine (C→A) mutant virus to directly establish whether a single or key m5C site is sufficient to mediate evasion of RIG‑I and inhibition of interferon responses.

Answer: We appreciate this valuable comment. We acknowledge that lacking our own bisulfite sequencing or m5C-RIP-seq data to verify specific m5C sites is a limitation of this work, and we have added relevant discussion of this limitation in the revised manuscript (page 22, lines 457-462). Notably, the previous work we cited (David G. Courtney et al., 2019, *Cell Host & Microbe*) also used the same HIV-1 NL4-3 strain and utilized PA-m5C-seq (a technique with higher sensitivity than m5C-RIP-seq) to map viral m5C modifications under NSUN2 knockout conditions. Their sequencing work specifically characterized m5C modifications located in the overlapping pol-vif region.

We did not generate mutant viruses carrying C-to-A substitutions at the six candidate cytosine sites for the following reason: mutations at five out of six cytosines will alter the amino acid sequences of Pol or Vif. Such changes may induce unknown functional defects in these viral proteins, disrupt native viral characteristics, and thereby confound the interpretation of viral infection phenotypes and immune responses.

To overcome this limitation, we synthesized m5C modified WT vif RNA and six individual single C-to-A mutant RNAs via in vitro transcription and carried out RNA pull-down assays. Our results show that single-point mutations at five cytosine residues substantially enhance the binding between viral RNA and RIG-I, while the C5098A mutation has mild influence on their interaction. These supplementary results have been fully presented and discussed in the revised manuscript (newly added Figure 6S; page 18, lines 362-371).

Most of the data are derived from immortalized cell lines, including 293T, HeLa, and Jurkat cells, whereas key conclusions are not validated in primary CD4⁺ T cells or macrophages. Validation in primary cells is strongly encouraged.

Answer: We appreciate this valuable suggestion. As recommended, we have validated our core phenotypes in two physiologically relevant cell systems: PMA-differentiated THP-1 macrophages and primary human CD4⁺ T cells.

First, THP-1 monocytes were differentiated into macrophages via PMA stimulation. Purified genomic RNA from WT or NSUN2-KO HIV-1 virions was transfected into these macrophages, and total cellular RNA was collected 24 hours after transfection to measure innate immune activation, with poly (I:C) set as the positive control. Consistent with results obtained from immortalized cell lines, viral RNA derived from NSUN2-KO virions induced substantially stronger innate immune responses (new Figure 4E-G; pages 12-13, lines 250-258).

Second, we infected primary human CD4⁺ T cells with authentic HIV-1 particles produced by WT or NSUN2 KO producer cells. Cellular RNA was extracted for qPCR analysis of *IFNA1*, *IFNB1* and viral *Gag* mRNA, while culture supernatants were harvested to measure viral production. In line with our findings in transformed cell lines, loss of NSUN2-dependent m5C methylation on viral RNA led to markedly enhanced innate immune signaling and severely impaired viral replication in primary CD4⁺ T cells (new Figure 5F-I; pages 14-15, lines 292-305).

The RIP assays presented in Figure 6 indicate that RIG‑I preferentially binds non‑m5C RNA; however, the mechanism by which m5C modification blocks RIG‑I‑RNA interaction remains unexplored. Structural modeling or biochemical approaches (e.g., EMSA with recombinant proteins) are recommended to address this question.

Answer: We appreciate this constructive suggestion. Following your recommendation, we performed EMSA experiments to biochemically characterize the binding between recombinant RIG-I protein and 5’ biotin-labeled RNA substrates. The gel shift results revealed that m5C-modified RNA exhibits weaker binding affinity to RIG-I protein compared with unmodified RNA (new Figure 6T; page 18, lines 371-376). Consistent observations were further validated by RNA pull-down assays. Moreover, RNA pull-down data show that single-point mutations at five cytosine residues substantially enhance the binding between viral RNA and RIG-I compare that of WT, while the C5098A mutation has mild influence on their interaction. All these newly acquired supporting data have been integrated into the revised manuscript (new Figure 6S; page 18, lines 362-371).

Given that m5C modification can influence RNA stability, splicing, or nuclear export, the authors should rule out the possibility that the reduced infectivity of NSUN2‑knockout viruses results from accelerated degradation of viral RNA or impaired translation efficiency.

Answer: We appreciate this thoughtful comment. We have carried out supplementary experiments to exclude the possibility that the reduced infectivity of NSUN2-knockout viruses results from accelerated viral RNA degradation or impaired viral protein translation.

To evaluate viral RNA stability, we performed actinomycin D chase assays to measure the decay rate of HIV-1 RNA. Briefly, WT, NSUN2-KO, and NSUN2-overexpressing cells were transfected with HIV-1 pNL4-3 plasmid. Twenty-four hours post-transfection, actinomycin D was supplemented to block de novo transcription, and viral mRNA was collected at indicated time points for quantification. Our results demonstrate that NSUN2-mediated m5C modification has no detectable influence on HIV-1 RNA stability (new Figure 1O-P; page 8, lines 143-154).

For translational efficiency detection, we focused on the m5C-containing vif transcript. Immunoblot results show that NSUN2 knockout in viral producer cell does not alter the expression level of Vif protein in HIV-1 infected cells. This evidence excludes impaired translation as a cause of reduced viral infectivity. These data have been added to the revised manuscript (new Figure 1N; page 8, lines 158-162).

**Minor Comments:**

The Western blot bands for p24 in Figure 1A lack quantitative densitometric analysis.

Answer: Thank you for pointing this out. Densitometric quantification of p24 bands has now been added to Figure 1A.

The authors state that HEK293T NSUN2 KO, shMDA5, and shRIG‑I cell lines were generated in‑house, but no validation data (e.g., sequencing, Western blot, or qPCR) are provided.

Answer: We appreciate your comment. The validation data for these cell lines have already been included in the original manuscript. Western blot validation of HEK293T NSUN2 KO cells is displayed in Figure 1, and RT-PCR verification of shMDA5 and shRIG-I cell lines can be found in Figure 6 respectively.

The background signal in the fluorescence images shown in Figure 1F appears elevated; adjustment of imaging conditions or quantification of signal‑to‑noise ratio is suggested.

Answer: We appreciate your careful comment. All four images in Figure 1F were reprocessed with consistent background correction to reduce non-specific background signals, and relevant image processing information has been added to the Figure 1F legend (line 486). Notably, these images are only for qualitative display, and all quantitative results are background-subtracted flow cytometry results shown in Figure 1G.
