# Supplementary material for: NSUN2-mediated m5C modification of HIV-1 RNA enables evasion of RIG‑I‑dependent innate immunity
Source: PLoS Pathog. 2026 Sep 25;22(9):e1014611. doi: 10.1371/journal.ppat.1014611 (PMC13614588; doi:10.1371/journal.ppat.1014611)
Supplement: Peer review history

# Peer review: Decision Letter 0

**DOI:** 10.1371/journal.ppat.1014611.r001

**Authors:** Richard Koup, Caijun Sun

10 May 2026

NSUN2-mediated m5C Modification of HIV-1 RNA Enables Evasion of RIG‑I‑Dependent Innate Immunity

PLOS Pathogens

Dear Dr. Zeng,

Thank you for submitting your manuscript to PLOS Pathogens. After careful consideration, we feel that it has merit but does not fully meet PLOS Pathogens's publication criteria as it currently stands. Therefore, we invite you to submit a revised version of the manuscript that addresses the points raised during the review process.

Please submit your revised manuscript by Jul 09 2026 11:59PM. If you will need more time than this to complete your revisions, please reply to this message or contact the journal office at plospathogens@plos.org. When you're ready to submit your revision, log on to https://www.editorialmanager.com/ppathogens/ and select the 'Submissions Needing Revision' folder to locate your manuscript file.

Please include the following items when submitting your revised manuscript:

* A letter that responds to each point raised by the editor and reviewer(s). You should upload this letter as a separate file labeled 'Response to Reviewers'. This file does not need to include responses to any formatting updates and technical items listed in the 'Journal Requirements' section below.

* A marked-up copy of your manuscript that highlights changes made to the original version. You should upload this as a separate file labeled 'Revised Manuscript with Track Changes'.

* An unmarked version of your revised paper without tracked changes. You should upload this as a separate file labeled 'Manuscript'.

As the corresponding author, your ORCID iD is verified in the submission system and will appear in the published article. PLOS supports the use of ORCID, and we encourage all coauthors to register for an ORCID iD and use it as well. Please encourage your coauthors to verify their ORCID iD within the submission system before final acceptance, as unverified ORCID iDs will not appear in the published article. Only the individual author can complete the verification step; PLOS staff cannot verify ORCID iDs on behalf of authors.

We look forward to receiving your revised manuscript.

Kind regards,

Caijun Sun

Academic Editor

PLOS Pathogens

Richard Koup

Section Editor

PLOS Pathogens

Sumita Bhaduri-McIntosh

Editor-in-Chief

PLOS Pathogens

orcid.org/0000-0003-2946-9497

Michael Malim

PLOS Pathogens

orcid.org/0000-0002-7699-2064

**Journal Requirements:**

If the reviewer comments include a recommendation to cite specific previously published works, please review and evaluate these publications to determine whether they are relevant and should be cited. There is no requirement to cite these works unless the editor has indicated otherwise.

1) Please ensure that the CRediT author contributions listed for every co-author are completed accurately and in full.

At this stage, the following Authors/Authors require contributions: Yuebo Xu, Mingyang Li, Yingying Song, Weili Kong, Jingjing Song, Jiali Zhang, Xinmu Xu, Haoyu Ma, Shun Yu, Shuliang Chen, and Cong Zeng. Please ensure that the full contributions of each author are acknowledged in the "Add/Edit/Remove Authors" section of our submission form.

The list of CRediT author contributions may be found here: https://journals.plos.org/plospathogens/s/authorship#loc-author-contributions

2) We ask that a manuscript source file is provided at Revision. Please upload your manuscript file as a .doc, .docx, .rtf or .tex. If you are providing a .tex file, please upload it under the item type u2018LaTeX Source Fileu2019 and leave your .pdf version as the item type u2018Manuscriptu2019.

3) We do not publish any copyright or trademark symbols that usually accompany proprietary names, eg ©,  ®, or TM  (e.g. next to drug or reagent names). Therefore please remove all instances of trademark/copyright symbols throughout the text, including:

- ® on page: 23

- TM on page: 23.

4) Please upload all main figures as separate Figure files in .tif or .eps format. For more information about how to convert and format your figure files please see our guidelines:

https://journals.plos.org/plospathogens/s/figures

5) Some material included in your submission may be copyrighted. According to PLOS’s copyright policy, authors who use figures or other material (e.g., graphics, clipart, maps) from another author or copyright holder must demonstrate or obtain permission to publish this material under the Creative Commons Attribution 4.0 International (CC BY 4.0) License used by PLOS journals. Please closely review the details of PLOS’s copyright requirements here: PLOS Licenses and Copyright. If you need to request permissions from a copyright holder, you may use PLOS's Copyright Content Permission form.

Please respond directly to this email and provide any known details concerning your material's license terms and permissions required for reuse, even if you have not yet obtained copyright permissions or are unsure of your material's copyright compatibility. Once you have responded and addressed all other outstanding technical requirements, you may resubmit your manuscript within Editorial Manager.

Potential Copyright Issues:

i) 7.: Please confirm whether you drew the images / clip-art within the figure panels by hand. If you did not draw the images, please provide (a) a link to the source of the images or icons and their license / terms of use; or (b) written permission from the copyright holder to publish the images or icons under our CC BY 4.0 license. Alternatively, you may replace the images with open source alternatives. See these open source resources you may use to replace images / clip-art:

- https://commons.wikimedia.org

- https://openclipart.org/

6) Please amend your detailed Financial Disclosure statement. This is published with the article. It must therefore be completed in full sentences and contain the exact wording you wish to be published.

1) State the initials, alongside each funding source, of each author to receive each grant. For example: "This work was supported by the National Institutes of Health (####### to AM; ###### to CJ) and the National Science Foundation (###### to AM)."

2) State what role the funders took in the study. If the funders had no role in your study, please state: "The funders had no role in study design, data collection and analysis, decision to publish, or preparation of the manuscript."

3) If any authors received a salary from any of your funders, please state which authors and which funders..

If you did not receive any funding for this study, please simply state: u201cThe authors received no specific funding for this work.u201d

**Reviewers' Comments:**

Reviewer's Responses to Questions

**Part I - Summary**

Please use this section to discuss strengths/weaknesses of study, novelty/significance, general execution and scholarship.

Reviewer #1: In this manuscript, Xu et al. report a novel and important mechanism by which HIV-1 hijacks the host m5C methyltransferase NSUN2 to install m5C modifications on viral genomic RNA, thereby blunting RIG-I–dependent innate immune sensing. The study is well conceived and thoughtfully executed. The data are solid, internally consistent, and supported by multiple complementary approaches, and the authors’ conclusions are well justified by the experimental evidence. Overall, the work significantly advances our understanding of how viral RNA epitranscriptomic regulation contributes to innate immune evasion and highlights NSUN2-dependent m5C modification as a potentially exploitable host pathway in antiviral immunity.

The manuscript is clearly written, the experimental logic is easy to follow, and the findings should be of broad interest to readers in viral pathogenesis, RNA biology, and innate immunity. I therefore consider this work suitable for publication in PLOS Pathogens after minor revision.

Reviewer #2: This manuscript investigates how HIV-1 uses a chemical modification called m5C on its RNA to hide from the host immune system. The authors show that a host enzyme, NSUN2, adds m5C marks to HIV-1 genomic RNA. These marks prevent the immune sensor RIG-I from detecting the viral RNA, which in turn reduces the production of type I interferons (IFN-I). When NSUN2 is knocked out in virus-producing cells, the resulting viruses carry less m5C on their RNA, trigger a stronger immune response, and replicate less efficiently. The authors further pinpoint RIG-I (not MDA5), and specifically its C-terminal domain (CTD), as the sensor that distinguishes between m5C-modified and unmodified viral RNA.

Overall, the topic is timely and the central findings are of interest. However, several experimental and conceptual concerns need to be addressed before this work can be considered for publication.

Reviewer #3: In this study, Yuebo Xu et al investigates the role of the NSUN2-mediated m5C Modification of HIV-1 RNA in modulating the innate immune response during viral infection. Building on previous findings that progeny virus is upregulated in response to NSUN2, the authors identify RIG-I, the sensor for non-m5C RNA, as preferentially binding non-m5C viral RNA. Through a combination of biochemical and virological assays, the study proposes a novel mechanism by which a HIV-1 utilizes m5C modification of viral genomic RNA to evade host-mediated innate immune regulation. They show that NSUN2-mediated m5C modification is sufficient to modestly evade innate immune activation and enhance viral replication both in vitro and in vivo, with approximately a 5-fold increase in viral load. While the premise is compelling, the rigor and depth of experimental validation should be improved to fully support the authors' conclusions.

Reviewer #4: This study reveals a novel immune evasion mechanism whereby HIV‑1 recruits the host methyltransferase NSUN2 to install m5C modifications on its genomic RNA, thereby shielding the viral RNA from RIG‑I recognition and suppressing the type I interferon response. These findings hold considerable significance for both fundamental research and translational applications. Nevertheless, the evidence supporting the core mechanistic model requires further strengthening, particularly with respect to the identification of functional m5C sites and the direct molecular basis of RIG‑I discrimination. The following key experiments are recommended to consolidate the conclusions.

**********

**Part II – Major Issues: Key Experiments Required for Acceptance**

Please use this section to detail the key new experiments or modifications of existing experiments that should be absolutely required to validate study conclusions.

Generally, there should be no more than 3 such required experiments or major modifications for a "Major Revision" recommendation. If more than 3 experiments are necessary to validate the study conclusions, then you are encouraged to recommend "Reject".

Reviewer #1: Major Comments

1.Previous studies have shown that NSUN2-mediated m5C modification regulates the stability of host RNAs. In the current study, the focus is on RIG-I sensing and immune evasion. Please clarify whether NSUN2-dependent m5C modification also affects HIV-1 RNA stability (e.g., RNA half-life/decay assays), and discuss how any stability effects might contribute to the observed phenotypes.

2.Most key experiments were performed in HEK293T, A549, and Jurkat cells. However, monocytes and macrophages are major physiological targets of HIV-1 and are highly relevant to innate immune sensing. Please validate the key findings in primary monocytes or monocyte-derived macrophages to confirm that NSUN2-mediated m5C modification promotes HIV-1 immune evasion in physiologically relevant cells.

Reviewer #2: 1. NSUN2 knockout affects many things beyond m5C on HIV-1 RNA. How can we be sure the effects are specific?

The study uses NSUN2 knockout as the main tool to study m5C’s role in immune evasion. The problem is that NSUN2 does not only modify HIV-1 RNA — it also adds m5C to many cellular RNAs, including tRNAs and mRNAs. Knocking out NSUN2 will therefore cause widespread changes in the cell (e.g., altered tRNA stability, translation, and gene expression), and some of these changes could indirectly affect viral replication or immune signaling.

The authors do include a good control in Figure 3D–E: they rescue NSUN2 KO cells with wild-type NSUN2 or a catalytically dead mutant and show that only the active enzyme restores normal IFN levels. However, this rescue is only done for cellular RNA. The same rescue experiment should be repeated in the HIV-1 infection context (i.e., in the experiments shown in Figures 4–6) to confirm that the immune evasion phenotype is truly due to NSUN2’s catalytic activity on viral RNA, rather than other indirect consequences of NSUN2 loss.

2. All experiments use immortalized cell lines

The study relies entirely on transformed cell lines (HEK293T, HeLa, A549, and Jurkat). While these are convenient and widely used, they do not fully represent the cells that HIV-1 naturally infects in the human body, such as primary CD4+ T cells and macrophages. These primary cells have different levels of RIG-I and MDA5, and their innate immune signaling can behave quite differently. To make the findings more physiologically relevant, the authors should validate at least the key results (e.g., stronger IFN induction by m5C-deficient virus, reduced viral replication) in primary CD4+ T cells or monocyte-derived macrophages.

3. The Vero vs. A549 comparison (Fig. 2H) does not cleanly demonstrate IFN-I dependence.

The authors compare viral infectivity in Vero cells (which cannot produce IFN) and A549 cells (which can) and conclude that the benefit of m5C modification depends on IFN-I signaling. The issue is that Vero and A549 cells are completely different cell types, so many factors beyond IFN competence could explain the observed differences.

A cleaner experiment would be to use the same cell type under two conditions: one where IFN signaling is intact, and one where it is blocked. This could be achieved by treating cells with an anti-IFNAR blocking antibody, a JAK inhibitor (e.g., ruxolitinib), or by using IFNAR-knockout cells alongside their wild-type counterparts.

Reviewer #3: 1.While the manuscript presents interesting and valuable findings, the overall clarity and flow would benefit from revision. I recommend improving the transitions between experiments to best guide the reader through the narrative. Clarifying the rationale behind each experimental step and providing more author-driven insights into data interpretation would enhance the coherence and readability of the manuscript.

For example, in lines 75-79, the authors introduce the concept of how NSUN2 mediates m5C modification and m5C sites without providing sufficient context regarding their prevalence or immunomodulatory significance, both within and beyond the specific context of NSUN2. Including this background would help readers better understand the relevance and implications of these elements as they pertain to this study.

2. Study Rigor - Descriptions of the study methodology should be expanded. It is unclear how HIV-1 infectivity(RLU) was processed to arrive at differential expression. How is the RIP experiment completed? What are the methods of the RIP experiment? The authors should provide details on how the data were calculated and normalized. Lastly, neither the figures nor the figure legends indicate sample sizes or whether the presented experiments have been reproduced independently,for example fig 1F,3A,6A.

3. In Figures 1H and 1I, the authors conclude that NSUN2 overexpression enhances viral replication. However, this conclusion is primarily based on the detection of HIV-1 infectivity(RLU) levels as an indicator of viral infection. Given the role of NSNUN2 on the regulation of progeny viruses, to substantiate the claim that NSNUN2 genuinely increases viral replication, it would be important to assess additional viral RNA or the production of infectious viral proteins to provide a direct and quantitative assessment of viral replication and infectivity. Additionally, as shown by the blue line, which represents RLU changes following NSUN2 KO, no significant difference were observed in Figures 1H and 1I, Is this consistent with the findings?

4.In Figure 2D, the values for the NSUN2 OE group are higher than those of the WT group, with poor reproducibility across three independent replicates. Furthermore, these results are inconsistent with the immune factor data presented in Figures 2A, 2B, and 2C. These data should be removed, or the experiment be repeated to further investigate this discrepancy.

5. In Figure 6, the RIP assay confirms the interaction between RIG-I and viral RNA. If this result is not supported by additional experiments, such as RNA pull-down or RNA FISH, this result raises some concerns for me.

Reviewer #4: Although the authors demonstrate global changes in m5C levels using NSUN2 knockout/overexpression and dot blot assays, direct evidence for specific m5C modification sites on HIV‑1 RNA (e.g., via bisulfite sequencing or m5C‑RIP‑seq) is lacking. The study merely cites previous work suggesting an m5C site in the overlapping Pol‑vif region, but this site has not been validated by sequencing in the HIV‑1 NL4‑3 background used herein. Moreover, the authors do not generate a site‑specific cytosine‑to‑adenine (C→A) mutant virus to directly establish whether a single or key m5C site is sufficient to mediate evasion of RIG‑I and inhibition of interferon responses.

Most of the data are derived from immortalized cell lines, including 293T, HeLa, and Jurkat cells, whereas key conclusions are not validated in primary CD4⁺ T cells or macrophages. Validation in primary cells is strongly encouraged.

The RIP assays presented in Figure 6 indicate that RIG‑I preferentially binds non‑m5C RNA; however, the mechanism by which m5C modification blocks RIG‑I‑RNA interaction remains unexplored. Structural modeling or biochemical approaches (e.g., EMSA with recombinant proteins) are recommended to address this question.

Given that m5C modification can influence RNA stability, splicing, or nuclear export, the authors should rule out the possibility that the reduced infectivity of NSUN2‑knockout viruses results from accelerated degradation of viral RNA or impaired translation efficiency.

**********

**Part III – Minor Issues: Editorial and Data Presentation Modifications**

Please use this section for editorial suggestions as well as relatively minor modifications of existing data that would enhance clarity.

Reviewer #1: Minor Comments

1.Statistical asterisks in the figures vary in size; please standardize them for readability.

2.In the Figure 2 legend, please correct NSUN KO to NSUN2 KO (line 356).

3.In the Materials and Methods, please correct ware to were (line 537).

Reviewer #2: 1. Typo in Figure 1 legend.

Line 346: “NSUN2-overexpressin” should be corrected to “NSUN2-overexpressing.”

2. Labeling error in Figure 7.

The schematic model labels the knockout condition as “NSU2N KO” — this should be “NSUN2 KO.”

3. The use of N1-methylpseudouridine in IVT RNA may confound interpretation.

In the in vitro transcription experiments, the authors replace UTP with N1-methylpseudouridine (N1-Me-Pseudo UTP; line 514). This modified nucleotide is itself well known to reduce innate immune activation (as noted in Ref. 41 of the manuscript). This raises a concern: if both the U and C positions are modified in the m5C-containing RNA, how can we confidently attribute the reduced IFN induction specifically to m5C? The authors should explain why they chose to use N1-methylpseudouridine and discuss whether this could complicate the interpretation of their m5C-specific results. Ideally, a parallel set of IVT RNAs using standard UTP (with and without m5CTP) should be included as a control.

Reviewer #3: 1.In Figure 1F and 2E,the images should add scale bars.

2. In Figure 6C and 6F,lanes should be labeled with sample names

3. Line 132 describes “in a does-dependent manner”,but Figure 2B do not show a clear dose-response relationship. Should this describe be revised or omitted?

Reviewer #4: The Western blot bands for p24 in Figure 1A lack quantitative densitometric analysis.

The authors state that HEK293T NSUN2 KO, shMDA5, and shRIG‑I cell lines were generated in‑house, but no validation data (e.g., sequencing, Western blot, or qPCR) are provided.

The background signal in the fluorescence images shown in Figure 1F appears elevated; adjustment of imaging conditions or quantification of signal‑to‑noise ratio is suggested.

**********

PLOS authors have the option to publish the peer review history of their article (what does this mean?). If published, this will include your full peer review and any attached files.

If you choose “no”, your identity will remain anonymous but your review may still be made public.

**Do you want your identity to be public for this peer review?**  For information about this choice, including consent withdrawal, please see our Privacy Policy.

Reviewer #1: No

Reviewer #2: No

Reviewer #3: No

Reviewer #4: No

**Figure resubmission:**

**Reproducibility:**

-->?>

# Author comment: Author response to Decision Letter 1

**DOI:** 10.1371/journal.ppat.1014611.r002

15 Jul 2026

Attachment Submitted filename: Response to Reviewers.docx

# Peer review: Decision Letter 1

**DOI:** 10.1371/journal.ppat.1014611.r003

**Authors:** Jason Brenchley, Caijun Sun

8 Sep 2026

Dear Prof. Zeng,

We are pleased to inform you that your manuscript 'NSUN2-mediated m5C Modification of HIV-1 RNA Enables Evasion of RIG‑I‑Dependent Innate Immunity' has been provisionally accepted for publication in PLOS Pathogens.

Before your manuscript can be formally accepted you will need to complete some formatting changes, which you will receive in a follow up email. A member of our team will be in touch with a set of requests.

Please note that your manuscript will not be scheduled for publication until you have made the required changes, so a swift response is appreciated.

IMPORTANT: The editorial review process is now complete. PLOS will only permit corrections to spelling, formatting or significant scientific errors from this point onwards. Requests for major changes, or any which affect the scientific understanding of your work, will cause delays to the publication date of your manuscript.

Should you, your institution's press office or the journal office choose to press release your paper, you will automatically be opted out of early publication. We ask that you notify us now if you or your institution is planning to press release the article. All press must be co-ordinated with PLOS.

Thank you again for supporting Open Access publishing; we are looking forward to publishing your work in PLOS Pathogens.

Best regards,

Caijun Sun

Academic Editor

PLOS Pathogens

Richard Koup

Section Editor

PLOS Pathogens

Sumita Bhaduri-McIntosh

Editor-in-Chief

PLOS Pathogens

orcid.org/0000-0003-2946-9497

Michael Malim

Editor-in-Chief

PLOS Pathogens

orcid.org/0000-0002-7699-2064

***********************************************************

Reviewer Comments (if any, and for reference):

Reviewer's Responses to Questions

**Part I - Summary**

Please use this section to discuss strengths/weaknesses of study, novelty/significance, general execution and scholarship.

Reviewer #1: (No Response)

Reviewer #2: (No Response)

Reviewer #3: (No Response)

**********

**Part II – Major Issues: Key Experiments Required for Acceptance**

Please use this section to detail the key new experiments or modifications of existing experiments that should be absolutely required to validate study conclusions.

Generally, there should be no more than 3 such required experiments or major modifications for a "Major Revision" recommendation. If more than 3 experiments are necessary to validate the study conclusions, then you are encouraged to recommend "Reject".

Reviewer #1: The authors have satisfactorily addressed all of my concerns.

Reviewer #2: (No Response)

Reviewer #3: (No Response)

**********

**Part III – Minor Issues: Editorial and Data Presentation Modifications**

Please use this section for editorial suggestions as well as relatively minor modifications of existing data that would enhance clarity.

Reviewer #1: The authors have satisfactorily addressed all of my concerns.

Reviewer #2: (No Response)

Reviewer #3: (No Response)

**********

PLOS authors have the option to publish the peer review history of their article (what does this mean?). If published, this will include your full peer review and any attached files.

If you choose “no”, your identity will remain anonymous but your review may still be made public.

**Do you want your identity to be public for this peer review?**  For information about this choice, including consent withdrawal, please see our Privacy Policy.

Reviewer #1: No

Reviewer #2: No

Reviewer #3: No

# Editor report: Acceptance letter

**DOI:** 10.1371/journal.ppat.1014611.r004

**Authors:** Jason Brenchley, Caijun Sun

Dear Prof. Zeng,

We are delighted to inform you that your manuscript, "NSUN2-mediated m5C Modification of HIV-1 RNA Enables Evasion of RIG‑I‑Dependent Innate Immunity," has been formally accepted for publication in PLOS Pathogens.

We have now passed your article onto the PLOS Production Department who will complete the rest of the pre-publication process. All authors will receive a confirmation email upon publication.

Thank you again for supporting open-access publishing; we are looking forward to publishing your work in PLOS Pathogens.

Best regards,

Dominique Soldati-Favre

Editor-in-Chief

PLOS Pathogens
